# Inactivation of SARS-CoV-2 by a chitosan/α-Ag_2_WO_4_ composite generated by femtosecond laser irradiation

**DOI:** 10.1038/s41598-022-11902-5

**Published:** 2022-05-17

**Authors:** Paula Fabiana Santos Pereira, Ana Carolina Alves de Paula e Silva, Bruna Natália Alves da Silva Pimentel, Ivo Mateus Pinatti, Alexandre Zirpoli Simões, Carlos Eduardo Vergani, Débora Ferreira Barreto-Vieira, Marcos Alexandre Nunes da Silva, Milene Dias Miranda, Maria Eduarda Santos Monteiro, Amanda Tucci, Carlos Doñate-Buendía, Gladys Mínguez-Vega, Juan Andrés, Elson Longo

**Affiliations:** 1 CDMF, LIEC, Department of Chemistry, Federal University of São Carlos (UFSCar), P.O. Box 676, São Carlos, SP 13565-905 Brazil; 2 Department of Physical and Analytical Chemistry, University Jaume I (UJI), 12071 Castelló, Spain; 3 Department of Dental Materials and Prosthodontics, School of Dentistry, São Paulo State University (UNESP), 1680 Humaitá Street, Araraquara, SP 14801-903 Brazil; 4 Faculty of Engineering of Guaratinguetá, São Paulo State University (UNESP), Guaratinguetá, SP 12516-410 Brazil; 5 Laboratory of Viral Morphology and Morphogenesis, Oswaldo Cruz Institute, Fiocruz, Avenida Brasil, Rio de Janeiro, Brazil; 6 Laboratory of Respiratory Viruses and Measles, Oswaldo Cruz Institute, Fiocruz, Avenida Brasil, Rio de Janeiro, Brazil; 7 GROC UJI, Institute of New Imaging Technologies, Universitat Jaume I, Avda. Sos Baynat sn, 12071 Castellón de la Plana, Spain; 8 Materials Science and Additive Manufacturing, University of Wuppertal, Gaußstr. 20, 42119 Wuppertal, Germany

**Keywords:** Diseases, Materials science

## Abstract

In the current COVID-19 pandemic, the next generation of innovative materials with enhanced anti-SARS-CoV-2 activity is urgently needed to prevent the spread of this virus within the community. Herein, we report the synthesis of chitosan/α-Ag_2_WO_4_ composites synthetized by femtosecond laser irradiation. The antimicrobial activity against *Escherichia coli*, Methicilin-susceptible *Staphylococcus aureus* (MSSA), and *Candida albicans* was determined by estimating the minimum inhibitory concentration (MIC) and minimal bactericidal/fungicidal concentration (MBC/MFC). To assess the biocompatibility of chitosan/α-Ag_2_WO_4_ composites in a range involving MIC and MBC/MFC on keratinocytes cells (NOK-si), an alamarBlue™ assay and an MTT assay were carried out. The SARS-CoV-2 virucidal effects was analyzed in Vero E6 cells through viral titer quantified in cell culture supernatant by PFU/mL assay. Our results showed a very similar antimicrobial activity of chitosan/α-Ag_2_WO_4_ 3.3 and 6.6, with the last one demonstrating a slightly better action against MSSA. The chitosan/α-Ag_2_WO_4_ 9.9 showed a wide range of antimicrobial activity (0.49–31.25 µg/mL). The cytotoxicity outcomes by alamarBlue™ revealed that the concentrations of interest (MIC and MBC/MFC) were considered non-cytotoxic to all composites after 72 h of exposure. The Chitosan/α-Ag_2_WO_4_ (CS6.6/α-Ag_2_WO_4_) composite reduced the SARS-CoV-2 viral titer quantification up to 80% of the controls. Then, our results suggest that these composites are highly efficient materials to kill bacteria (*Escherichia coli*, *Methicillin-susceptible Staphylococcus aureus*, and the yeast strain *Candida albicans*), in addition to inactivating SARS-CoV-2 by contact, through ROS production.

## Introduction

Currently, the worldwide emergence and rapid evolution of the COVID-19 infectious disease induced by the SARS-CoV-2 virus is an ever-growing global crisis^1^. Despite the development of several vaccines and extensive vaccination programs, there is an urgent need to discover novel materials and strategies to combat and prevent the spread of viral infections for dealing with current and future pandemics. In this unprecedented scenario, materials capable of killing pathogens (such as bacteria, fungi and viruses) are highly desirable in applications requiring a protective barrier against contamination, transmission, and proliferation^2–5^.

In this frenetic race against COVID-19, our research group has contributed significantly by developing new technologies based on the synthesis of potent biocidal materials using substantial cumulative knowledge rapidly translated to various multi-tasking applications, such as personal protective equipment (gloves, face masks, clothing, etc.) and devices for disinfection of surfaces/surroundings. In particular, different complex silver-based oxides with potent antibacterial and antifungal activities have been presented, such as Ag_2_CrO_4_^6,7^, the three polymorphs of Ag_2_WO_4_^8,9^, Ag_3_PO_4_^10,11^, α-AgVO_3_^12^ and β-Ag_2_MoO_4_^13^. To provide a deeper understanding and establish a correlation among morphology, surface energy and biocidal activity, first principles calculations were conducted at the density functional theory (DFT) level to complement and rationalize the experimental findings^14^. In addition, the formation of Ag nanoparticles (AgNPs), on α-Ag_2_WO_4_ induced by femtosecond (fs) laser irradiation has been proved. It was found that the as-synthetized composite displays a 32-fold improvement in the bactericidal properties in relation to α-Ag_2_WO_4_^15,16^. On the other hand, very recently different materials with antiviral and bactericidal properties were reported: (i) a SiO_2_-Ag composite as a highly virucidal material to be applied in polycotton fiber surfaces for the prevention of viral proliferation and transmission of SARS-CoV-2^17^, (ii) immobilized SiO_2_-Ag composites in a polymeric matrix (ethyl vinyl acetate) with highly antibacterial activity towards *Escherichia coli* and SARS-CoV-2^18^, and (iii) nanostructured aluminum Al 6063 alloy surfaces to inactivate the SARS-CoV-2^19^. These works open up an innovative path for the design and application of adequate technologies to the next generation of antiviral surfaces in order to combat SARS-CoV-2.

Fs laser irradiation provides unique opportunities to investigate and control the behavior of materials under strong electronic, thermal, and mechanical non-equilibrium conditions^20^. High-intensity pulsed laser of a colloidal dispersion of nano or microparticles can provoke size reduction^21^ by Coulomb explosion and/or particle surface melting depending on the pulse duration and intensity^20,22–25^. This methodology is more advantageous than wet chemical methods because when used to modify or functionalize materials for biomedical applications, it avoids the presence of additional substances capable of decreasing the materials biocompatibility.

Chitosan (CS) is a natural polysaccharide with alkaline substances extracted from chitin shells of crustaceans. Its structure contains lots of free amino (–NH_2_), and hydroxy (–OH) groups, which are favorable to various chemical modifications and hybridization. So far, CS has many important advantages, such as ease of synthesis, low cost, non-toxicity, high biocompatibility and biodegradability^26^, as well as inherent antibacterial, antifungal, adsorbent^27–29^ and bioactivity properties^30^. Due to such features, CS and its composites have been widely used as drug release agents^31^, skin substitutes^32^, vaccine delivery agents^33^, antibacterial agents^34–37^, wound dressings^36,38,39^, and wastewater treatment agents^40^. Moreover, it is well known that CS is an efficient host for stabilizing metal oxides, such as TiO_2_ so as to enhance the mechanical, physical, and biological properties of the CS biopolymer^41–44^, as the TiO_2_ agglomeration would mask its ability to be used in technological applications.

Taking into account the above considerations, the motivation for this work is derived from our previous studies. Considering that CS is capable of stabilizing Ag nanoparticles formed during fs laser irradiation, herein we selected an appropriate processing route to obtain CS/α-Ag_2_WO_4_ composites with enhanced biocidal activity. As this procedure provides nontoxicity, low cost, good thermal stability and excellent biocompatibility, it can be considered a frontline measure to prevent COVID-19 disease, thus representing the focus of the present work. Our aims can be summarized as follows: (i) the synthesis of this composite; (ii) the characterization of the photochemical properties and exploration of the potential cooperative effects resulting from the combination of the two mentioned functional entities; and finally (iii) the evaluation of the antimicrobial (against *Escherichia coli*, *Methicillin-susceptible Staphylococcus aureus*, and the yeast strain *Candida albicans*) and SARS-CoV-2 antiviral activities of the synthesized CS/α-Ag_2_WO_4_ composite. The α-Ag_2_WO_4_ was synthesized by the coprecipitation method and dispersed in three CS concentrations (3.3, 6.6 and 9.9 g/L). The suspension formed was then irradiated by fs laser. The as-obtained composites were denoted as CS3.3/α-Ag_2_WO_4_, CS6.6/α-Ag_2_WO_4_ and CS9.9/α-Ag_2_WO_4_, respectively.

## Results and discussion

### Structural analysis

The α-Ag_2_WO_4_ was synthesized by the coprecipitation method as previously reported^45–47^ (see SI for experimental details). Figure S2A shows the X-ray diffraction (XRD) patterns of the α-Ag_2_WO_4_ microcrystal, confirming its orthorhombic structure according to the inorganic crystal structure database (ICSD)—card no. 4165^48^. This figure also presents the XRD peaks related to the semi-crystalline structure of the CS polymer, showing intense peaks located at 11.4°, 21.0° and 22.5°. Figure S2B displays the XRD patterns of the CS/α-Ag_2_WO_4_ composites irradiated by fs laser, where a characteristic XRD pattern of an amorphous phase related to the CS polymer and peaks assigned to the α-Ag_2_WO_4_ orthorhombic structure can be observed. According to Khan et al.^49^, the crystallinity of the CS polymer is ascribed to the number of –NH_2_ and –OH groups in the structure. These groups form strong intra and intermolecular hydrogen bonds, leading to a certain regularity of the CS structure, thus resulting in the appearance of crystalline regions. The intensity of the CS XRD peaks decrease for the CS/α-Ag_2_WO_4_ composites (Fig. S2B), which was also observed by Khan et al.^49^. Moreover, the inset in Fig. S2B shows an increase in peak intensity at 2*θ* = 28.8° for all composites, indicating a preferential growth of the α-Ag_2_WO_4_ structure towards the (3 0 1) crystallographic plane.

Fourier-transform infrared spectroscopy (FTIR) was carried out to evaluate the vibrational modes of the as-synthesized CS/α-Ag_2_WO_4_ composites. Figure S3 displays the characteristics bands of the CS polymer. Bands related to the (–C=O) carbonyl group (2000–1650 cm^−1^) cannot be observed, indicating no degradation of the CS polymer after fs laser irradiation^49^. For the CS/α-Ag_2_WO_4_ composites, the CS polymer bands present lower intensity. In addition, bands ascribed to [WO_4_^2−^] and [AgO_x_] (x = 2, 4, 6, and 7) can be considered constituent clusters of the α-Ag_2_WO_4_ structure (Fig. S4).

### Morphological analysis

**Figure 1:**
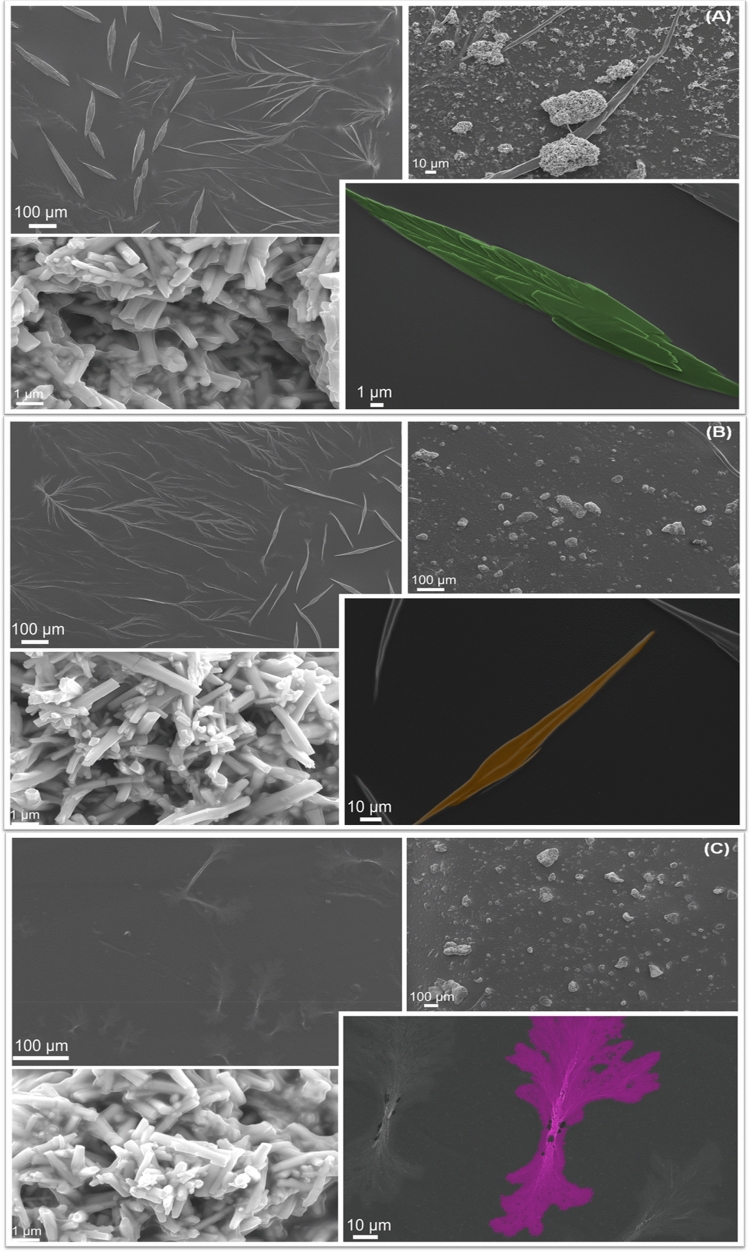
FE-SEM images of (**A**) CS3.3/α-Ag_2_WO_4_, (**B**) CS6.6/α-Ag_2_WO_4_, and (**C**) CS9.9/α-Ag_2_WO_4_ composites irradiated by fs laser.

Figure 1A–C show field emission scanning electron microscopy (FE-SEM) images of the CS/α-Ag_2_WO_4_ composites, where it can be seen that the CS polymers with α-Ag_2_WO_4_ microcrystals are dispersed under and over the film. Several α-Ag_2_WO_4_ irregular rod-like structures with hexagonal face of several sizes can be observed for all samples^50,51^, besides a covering around each microcrystal formed by the CS polymer. In the CS film, it is possible to note a ribbing forming root-type structures and different morphologies, such as pointed spear-like structures, which differ in shape depending on the CS solution concentration. While for the CS3.3/α-Ag_2_WO_4_ composite roughness layers can be observed on the surface (Fig. 1A), for the CS6.6/α-Ag_2_WO_4_ composite the surface is, smoother (Fig. 1B). In contrast, for the CS9.9/α-Ag_2_WO_4_ composite, different roots can be found forming a foliage-type structure (Fig. 1C). Energy-dispersive spectroscopy (EDS) analysis performed on the α-Ag_2_WO_4_ rod-like structures confirmed the presence of the elements Ag, W and O. On the other hand, only Ag was observed in the ribbing, root and spear-like structures (Fig. S5). These structures were formed due to fs laser beam and the effect of CS polymer, which promoted the reduction of Ag^+^ to Ag^0^ (AgNPs)^15,30,52–58^.

**Figure 2:**
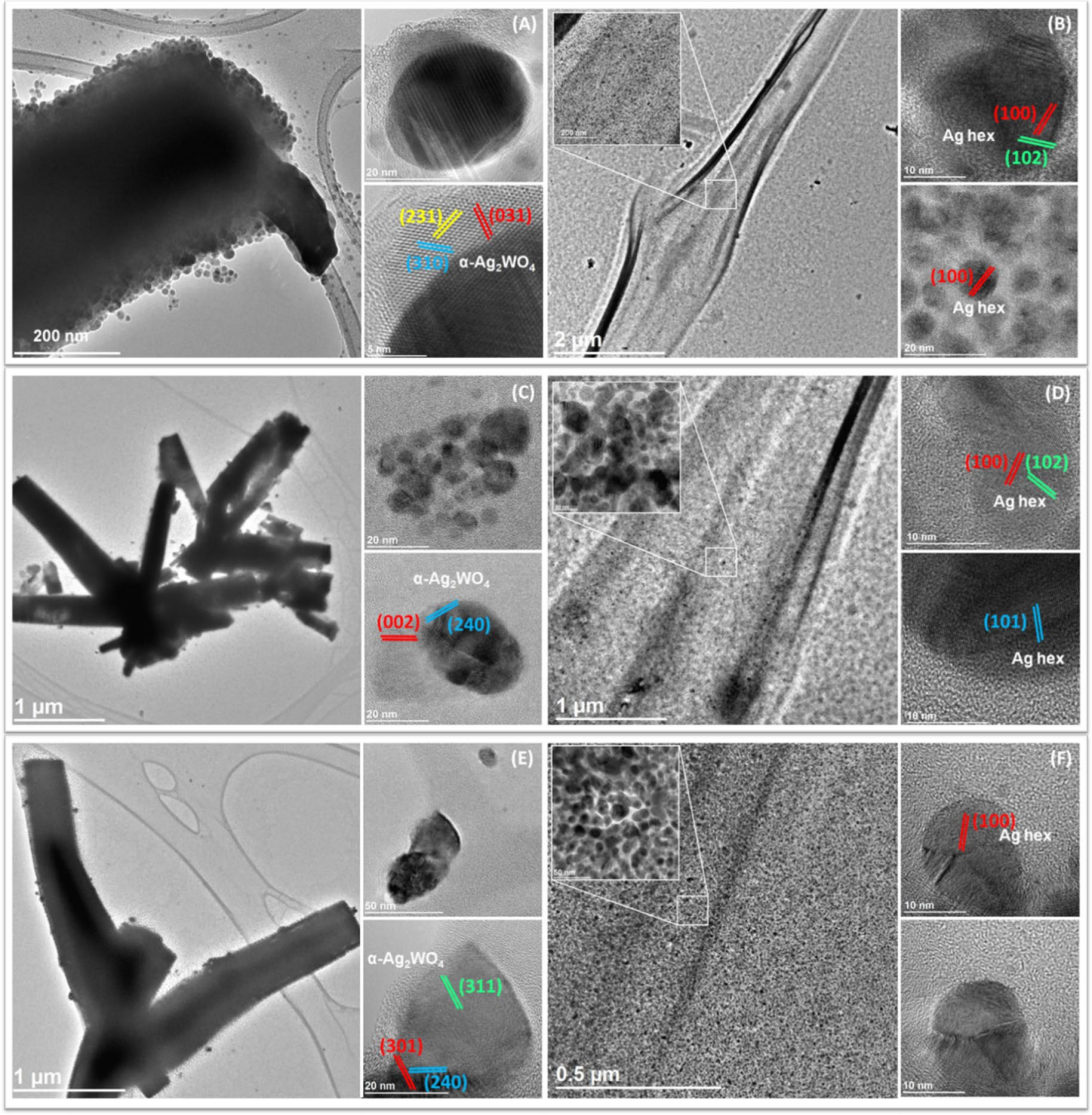
(**A**, **C**, **E**) TEM and HR-TEM images of α-Ag_2_WO_4_ rods and α-Ag_2_WO_4_ NPs; and (**B**, **D**, **F**) TEM and HR-TEM images of AgNPs related to CS3.3/α-Ag_2_WO_4_, CS6.6/α-Ag_2_WO_4_ and CS9.9/α-Ag_2_WO_4_ composites, respectively.

Figure 2 shows the transmission electron microscopy (TEM) and high-resolution transmission electron microscopy (HR-TEM) images of the CS/α-Ag_2_WO_4_ composites. The particles formed on the surface of the CS polymer are composed of larger α-Ag_2_WO_4_ NPs and smaller AgNPs, as proved by HR-TEM and EDS analyses. According to Murugadoss et al.^59^, the AgNPs are stabilized due to the excess of –NH_2_ and –OH groups present in the CS polymer chain (Fig. S6A–C).

The α-Ag_2_WO_4_ structure was confirmed in the CS3.3/α-Ag_2_WO_4_ composite by indexing the (310), (031) and (231) planes with interplanar distances of 3.45, 3.31 and 2.82 Å, respectively (Fig. 2A). For the CS6.6/α-Ag_2_WO_4_ composite, the identified planes were (002) and (240) with interplanar distances of 2.95 and 2.62 Å, respectively (Fig. 2C). Finally, for the CS9.9/α-Ag_2_WO_4_ composite, the indexed planes were (301), (311) and (240) with interplanar distances of 3.08, 2.98 and 2.62 Å, respectively (see Fig. 2E), according to the JCPDS database (PDF34-61)^48^. The HR-TEM images of the AgNPs on the CS/α-Ag_2_WO_4_ composites correspond to the typical hexagonal structure and are indexed to the (100), (101) and (102) planes with interplanar distances of 2.50, 2.42 and 2.23 Å, respectively, according to JCPDS database (PDF87-598) (Fig. 2B,D,F)^16,60^.

The presence of the –OH and –NH_2_ groups in the CS polymer leads to various chemical bonds with metals^27–29^, causing it to act as a chelating agent. However, in an acetic acid (AA) medium, the CS polymer reacts with H^+^ ions to produce protonated CS with –NH_3_^+^ functional groups. Thus, the CS polymer provides free electrons to reduce Ag^+^ to Ag^0^^30,49,55–58,61,62^, which was also demonstrated for Cu and Au^30,49,55–58,61,62^, resulting in the formation of α-Ag_2_WO_4_ NPs and AgNPs with near-spherical morphologies due to fs laser irradiation.

### Biocidal analysis

#### Evaluation of the minimum inhibitory concentration (MIC) and minimum fungicidal/bactericidal concentration (MFC/MBC)

Initially, no statistical difference between the data obtained from vehicles (CS and AA) and the control (CT) without treatment (data not shown) was observed, which means that the vehicles did not interfere with the viability of the microorganisms tested and that the activity observed was solely due to fs laser irradiation of the CS/α-Ag_2_WO_4_ composites. The literature describes that pure CS (capping agent), as well as AA (used to dissolve CS), has inhibitory activity against different species of microorganisms^63,64^. However, it was verified that when used in low quantity these vehicles did not present antimicrobial activity.

In a previous study, our research group^15^ showed that α-Ag_2_WO_4_ irradiated by fs laser can increase its biocidal activity when compared to the α-Ag_2_WO_4_. In this work, it was observed that the antimicrobial activity was dependent on the CS concentration. The MIC was determined by visual inspection (Table 1). It was found that the CS3.3/α-Ag_2_WO_4_ and CS6.6/α-Ag_2_WO_4_ composites presented similar activity (MIC ranging from 0.49 to 1.95 µg/mL) for all microorganisms tested, while the CS9.9/α-Ag_2_WO_4_ composite increased onefold against the microorganisms *S. aureus* and *C. albicans* (3.9 and 0.98 µg/mL, respectively). The MFC/MBC were determined for the activity capable of inhibiting 99.9%. Therefore, it was possible to observe a concentration increase in relation to the MIC necessary to reach more than 90% inhibition. This increase was represented as “fold change” in Table 1, which displays the MIC value, together with the MFC/MBC values and their respective inhibition index (%) according to CFU/mL normalized by the CT group.

**Table 1:** MIC (µg/mL) and MFC/MBC (µg/mL) of CS/α-Ag_2_WO_4_ composites irradiated by fs laser in three different CS concentrations (g/L) against the microorganisms *C. albicans, S. aureus* and *E. coli,* and the inhibition index (%) calculated according to CFU/mL data and normalized by control without treatment (n = 8).

Chitosan (g/L)	3.3			6.6			9.9			
Microorganisms	MIC	MFC/MBC	Fold change	MIC	MFC/MBC	Fold change	MIC	MFC/MBC	Fold change	
*C. albicans*	1.95 (12)	7.81 (100)	2	1.95 (23.6)	7.81 (100)	2	3.9 (20.9)	31.25 (100)	3	µg/mL (%)
*S. aureus*	0.49 (12.6)	3.9 (100)	3	0.49 (14.8)	1.95 (100)	2	0.98 (24.1)	7.81 (100)	3	
*E. coli*	0.49 (100)	0.49 (100)	0	0.49 (100)	0.49 (100)	0	0.49 (100)	0.49 (100)	0	

As a consequence, all CS/α-Ag_2_WO_4_ composites irradiated by fs laser were more effective against Gram-negative (*E. coli*) than Gram-positive (*S. aureus*) bacteria and the yeast (*C. albicans*). This difference can be explained by the structure of the cell wall, which in the Gram-positive bacteria and the yeast is composed of peptidoglycan and teichoic acid, bringing more stability to the cell wall. Studies suggest that silver ions are able to attach to the membrane surface of the microorganism, leading to membrane disruption and increasing its permeability. As a result, they could enter cells, condensing DNA and reacting with proteins. Moreover, thiol groups, which are responsible for enzyme activity, are inactivated by reacting with silver^64,65^.

Accordingly, a greater efficacy was also observed for the *E. coli*, a Gram-negative bacteriuma. Its membrane is composed of lipopolysaccharides (LPS) containing phosphate and pyrophosphate groups that make the cell surface negatively charged. Additionally, as CS is a cationic polymer it facilitates the binding of ions to the membrane, causing the microorganism inactivation, as previously described^55^. In another study with *E. coli*, researchers reported that silver ions trigger the separation of DNA strands and weaken the link between protein and DNA, thus altering vital processes for the microorganism^66^. Therefore, the CS6.6/α-Ag_2_WO_4_ composite was considered to have the best antimicrobial activity, as it exhibited low concentrations for the microbicidal effect and still presented a little difference between the MIC and MFC/MBC values (low fold-change value).

#### Cytotoxicity analysis

In this section we evaluate the cytotoxicity of CS/α-Ag_2_WO_4_ composites irradiated by fs laser with the aim of developing an agent with antimicrobial activity for biomedical application. According to the literature, CS solutions exhibit toxicity depending on the dose and synthesis method^67^. Jena et al.^68^ observed that the cytotoxicity of CS with AgNPs (CS-AgNPs) is dose-dependent and that the cell viability decreases as the concentration increases. The same cell behavior was observed in this study with CS/α-Ag_2_WO_4_ composites irradiated by fs laser. However, the authors did not find any decrease in cell viability when evaluating only CS^68^, which may be explained by the CS synthesis method.

Herein, the cytotoxicity profile of different concentrations of CS and AA vehicles was evaluated in the NOK-si lineage cell by 3-(4,5-dimethyl-2-thiazolyl)-2,5-diphenyl-2*H*-tetrazolium bromide (MTT) and Alamar Blue assay. In the analysis by MTT assay (Fig. S7), it was observed that after 24 h of contact (Fig. S7A,D,G,J) all dilutions (C1–C7) presented statistical difference in relation to CT, except the C6 dilution for CS9.9 (Fig. S7G), and C5, C6 and C7 dilutions for AA (Fig. S7J). After 48 h (Fig. S7B,E,H,K) and 72 h (Fig. S7C,F,I,L), it was possible to observe that the cytotoxicity profile of all vehicles changed. No statistical difference in relation to CT of the CS vehicle in the lowest dilutions and in any dilution of the AA vehicle was noted. In general, we observed that the AA vehicle did not present a cytotoxic profile and that the CS6.6 vehicle presented the minimal cytotoxic dilutions for the MTT assay.

**Figure 3:**
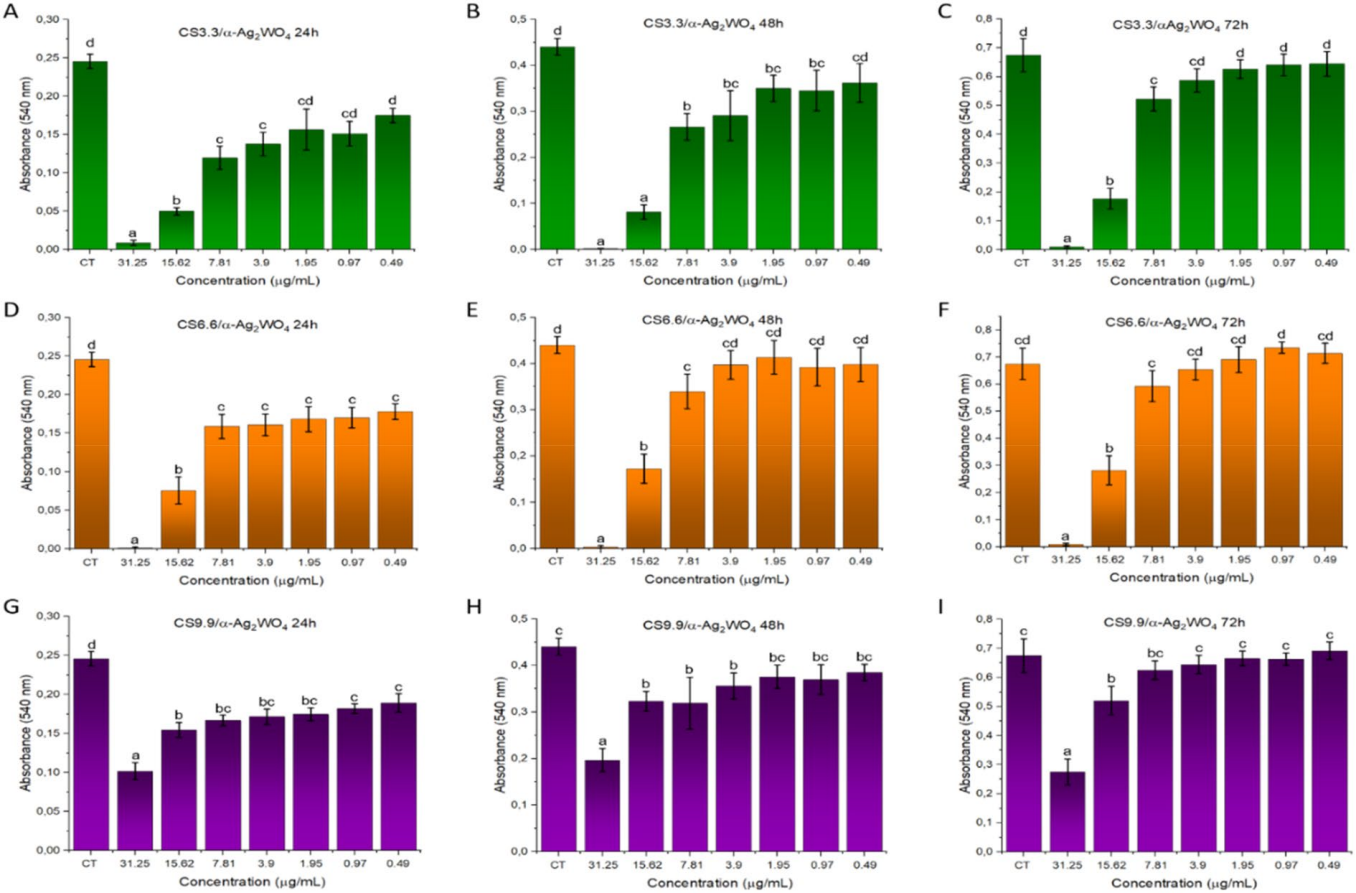
Cytotoxity profile by MTT assay. Mean absorbance values after 24 h (**A**, **D**, **G**), 48 h (**B**, **E**, **H**) and 72 h (**C**, **F**, **I**) of treatment with different concentrations of CS3.3/α-Ag_2_WO_4_ (**A**, **B**, **C**), CS6.6/α-Ag_2_WO_4_ (**D**, **E**, **F**) and CS9.9/α-Ag_2_WO_4_ (**G**, **H**, **I**) composites. CT: control; CS: chitosan. Different letters denote statistically significant differences between concentrations (*p* < 0.05), (n = 12).

The cytotoxicity profile analysis by MTT assay of the CS/α-Ag_2_WO_4_ composites was carried out at different concentrations (Fig. 3). The results revealed that after 24 h (Fig. 3A,D,G) all composites presented statistical difference in relation to CT, with exception of the CS3.3/α-Ag_2_WO_4_ composite (Fig. 3A), which showed no statistical difference in relation to CT at the three last concentrations (1.95–0.49 µg/mL). However, after 72 h (Fig. 3C,F,I) the cytotoxicity profile of the composites experienced some changes. No statistical difference in relation to CT was observed in the lowest concentrations for CS6.6/α-Ag_2_WO_4_ and CS9.9/α-Ag_2_WO_4_ (7.8–0.49 µg/mL), as well as for CS3.3/α-Ag_2_WO_4_ (3.9–0.49 µg/mL). We can then conclude that the CS6.6/α-Ag_2_WO_4_ composite presented the best non-cytotoxic profile since the vehicle CS6.6 did not show cytotoxicity for the last 5 dilutions (C3–C7), corresponding to 7.81–0.49 µg/mL concentrations of the CS6.6/α-Ag_2_WO_4_ composite, which also presented no cytotoxicity.

In contrast, when the cytotoxicity profile of the CS and AA vehicles was evaluated by Alamar Blue assay (Fig. S8), we did not observe any statistical difference in relation to CT, independent of the incubation time, which means that the vehicles did not present any cytotoxic profile.

**Figure 4:**
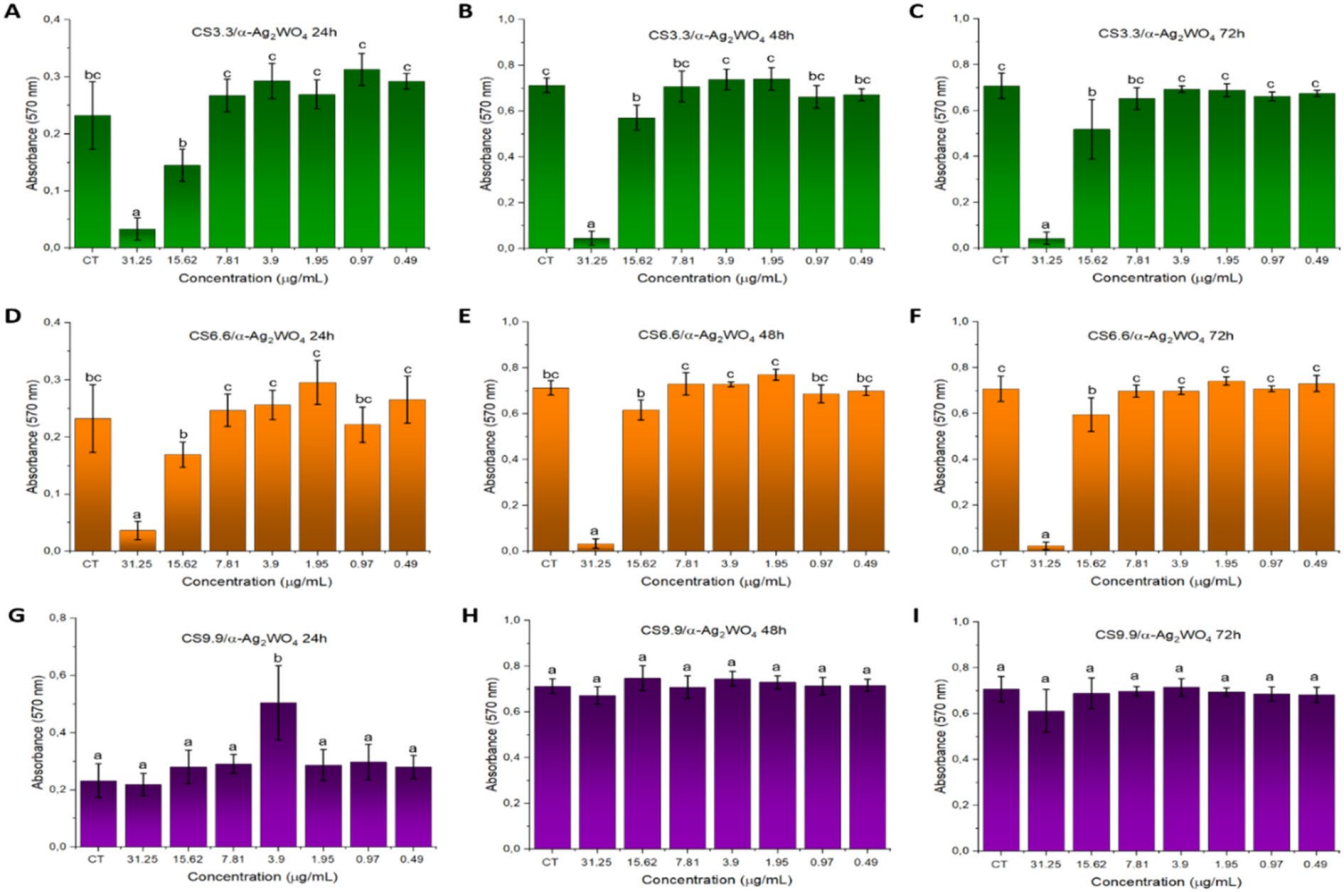
Cytotoxicity profile by Alamar Blue assay. Mean absorbance values after 24 h (**A**, **D**, **G**), 48 h (**B**, **E**, **H**) and 72 h (**C**, **F**, **I**) of treatment with different concentrations of CS3.3/α-Ag_2_WO_4_ (**A**–**C**), CS6.6/α-Ag_2_WO_4_ (**D**–**F**) and CS9.9/α-Ag_2_WO_4_ (**G**–**I**) composites. CT: control; CS: chitosan. Different letters denote statistically significant differences between concentrations (*p* < 0.05), n = 12.

When the cytotoxicity profile of the CS/α-Ag_2_WO_4_ composites was analyzed at different concentrations by the Alamar Blue assay (Fig. 4), it was noted that after 24 h of contact only the 31.25 µg/mL concentration for the CS3.3/α-Ag_2_WO_4_ and CS6.6/α-Ag_2_WO_4_ composites presented statistical difference (Fig. 4A, D), which was maintained for 48 and 72 h, respectively. For the CS9.9/α-Ag_2_WO_4_ composite, no statistical difference was observed after 24 h, 48 or 72 h of contact (Fig. 4G–I). Assis et al.^15^ also reported a non-cytotoxic effect by the Alamar Blue assay when similar concentrations of fs-irradiated α-Ag_2_WO_4_ microcrystals were maintained in contact with cells for 24 h, even in the highest concentration tested (31.25 µg/mL). Despite the fact that the authors evaluated a different cell line, the CS concentration apparently had an influence on the biological activity. For the CS3.3/α-Ag_2_WO_4_ composite, after 48 and 72 h of contact (Fig. 4B, C), it was found that the cytotoxicity profile presented a small change in the statistical difference for the 15.62 µg/mL concentration. On the other hand, for the CS6.6/α-Ag_2_WO_4_ composite the same concentration showed statistical difference only after 72 h (Fig. 4F). Therefore, we can infer that the CS6.6/α-Ag_2_WO_4_ composite presented the best non-cytotoxic profile, as it maintained the profile of less cytotoxicity for longer time, considering that any dilution of the CS6.6 vehicle showed cytotoxicity.

Although in both methods the reagents are metabolized by mitochondrial enzymes and present in the cytoplasm, it is known that the MTT reagent is more metabolized by mitochondrial enzymes than the Alamar blue assay, thus providing important information on the influence of the compound on the cell^69^. Through data analysis it was possible to observe that the Alamar blue assay showed high viability index higher concentrations. These indexes were stable during the three incubation times, demonstrating that the composites did not damage the pathway through which the Alamar blue reagent is metabolized^70^. In contrast, the MTT assay showed low viability index at lower concentrations in the first 24 h of treatment, whereas in the periods of 48 and 72 h it was possible to observe a small cell recovery, which increased the viability index at higher concentrations, reaching the concentrations observed in the Alamar blue assay after 72 h of incubation. This profile suggest that the composites initially induce a stress in the mitochondrial metabolism without causing any damage to cell, as evidenced by their recovery. Therefore, we believe that it is important to show the results of both methodologies, elucidating that despite the oxidative stress generated (which is already known by the microcrystal), this is not a determinant for the loss of cell viability.

#### SARS-CoV-2 inactivation by the CS6.6/α-Ag_2_WO_4_ composite

The 4.0 µg/mL concentration of the CS6.6/α-Ag_2_WO_4_ composite was selected for SARS-CoV-2 antiviral assays due to its greatest efficiency in the inhibition of bacterial and fungal growth, as well as its best non-cytotoxic profile.

**Figure 5:**
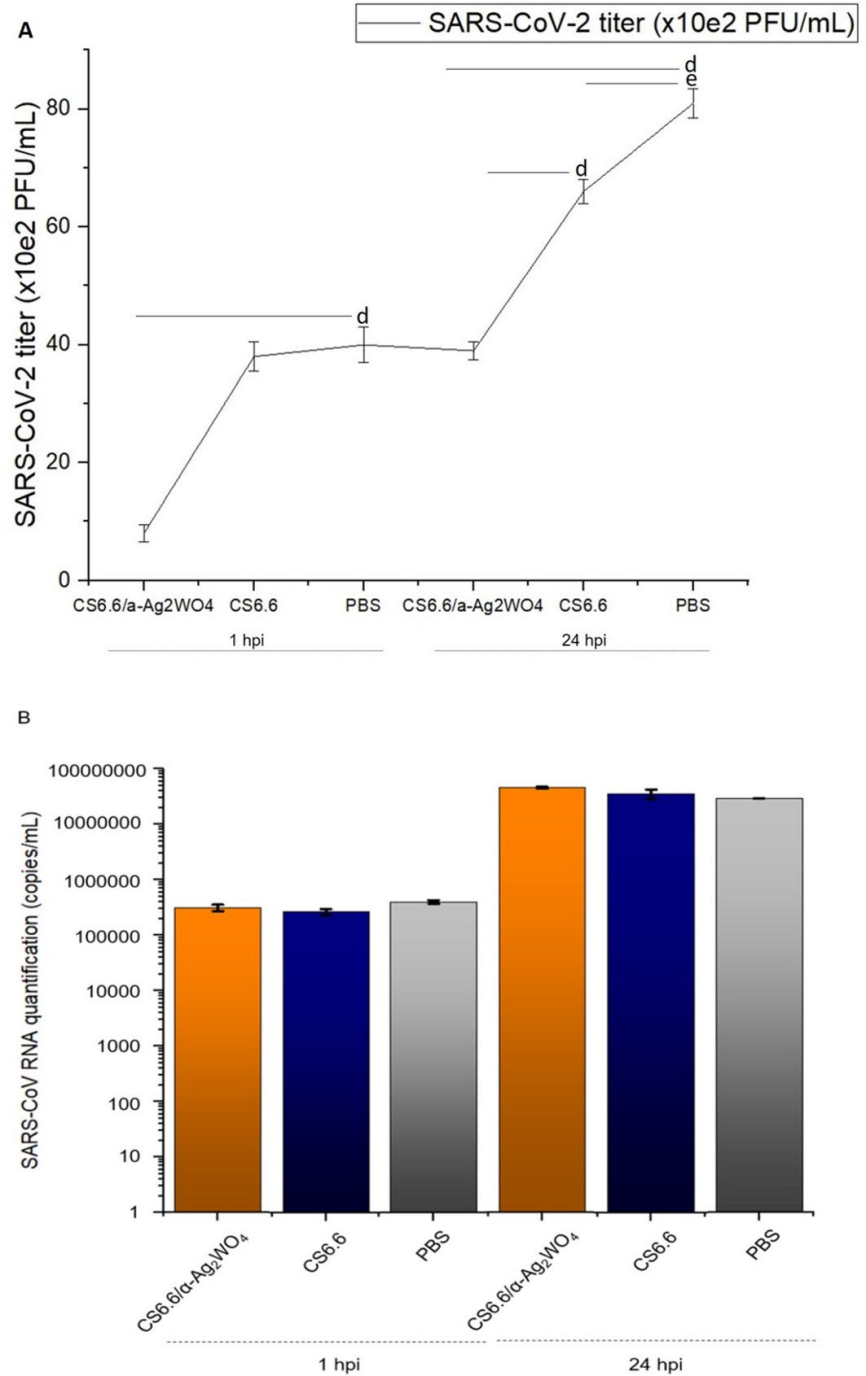
The CS6.6/α-Ag_2_WO_4_ composite treatment inactivate SARS-CoV-2 virus. Vero-E6 cells were infected with virus exposed to CS6.6/α-Ag_2_WO_4_ (4.0 µg/mL), CS6.6 (4.0 µg/mL) or PBS. The supernatants were harvested 1 and 24 hpi and the virus titer analyzed by PFU/mL (**A**) and RNA quantified by qRT-PCR (**B**). n = 3, ^d^*p* < 0.005, ^e^*p* < 0.05.

The viral titer in the cell supernatants was quantified by PFU/mL assay at 1 and 24 hpi (hours post infection) to study the effect of the CS6.6/α-Ag_2_WO_4_ composite against virus inactivation (Fig. 5). SARS-CoV-2 titer at 1-hpi supernatants was equivalent to 4.0 × 10^3^ PFU/mL when the cells were incubated with the virus exposed to CS6.6 or PBS, used as controls. Initially, the CS6.6/α-Ag_2_WO_4_ composite reduced the viral titer quantification to 0.8 × 10^3^ PFU/mL, an inhibition of virus infection in 80% of the controls. After 24 h of exposure to the inactivated virus solution with the CS6.6/α-Ag_2_WO_4_ composite, the viral titer quantified in the cell culture supernatant was 41% and 52% lower than that quantified in the cell culture supernatants from exposure to the inactivated virus solution with CS6.6 and PBS, respectively (Fig. 5A). This reduction in the viral titer may reflect the SARS-CoV-2 virucidal effect promoted by the CS6.6/α-Ag_2_WO_4_ composite. Despite this reduction, the number of virus RNA copies recovered from the infected cells under different treatments were not changed either at 1 or 24 hpi (Fig. 5B). These results indicate that the exposure to the CS6.6/α-Ag_2_WO_4_ composite inactivated viral infection even though the identification of the pathogen through its genes was enabled. CS products such as cationically modified derivatives can inhibit human coronavirus replication^71^, and this inhibitory effect was observed in viral titer quantification from cell culture supernatant exposed to CS6.6 24 hpi (Fig. 5A).

### Morphological analysis by TEM of Vero-E6 cell cultures infected with SARS-CoV-2 untreated and treated with CS6.6/α-Ag_2_WO_4_

#### Vero-E6 cells untreated and treated with CS6.6/α-Ag_*2*_WO_4_ composite (controls)

**Figure 6:**
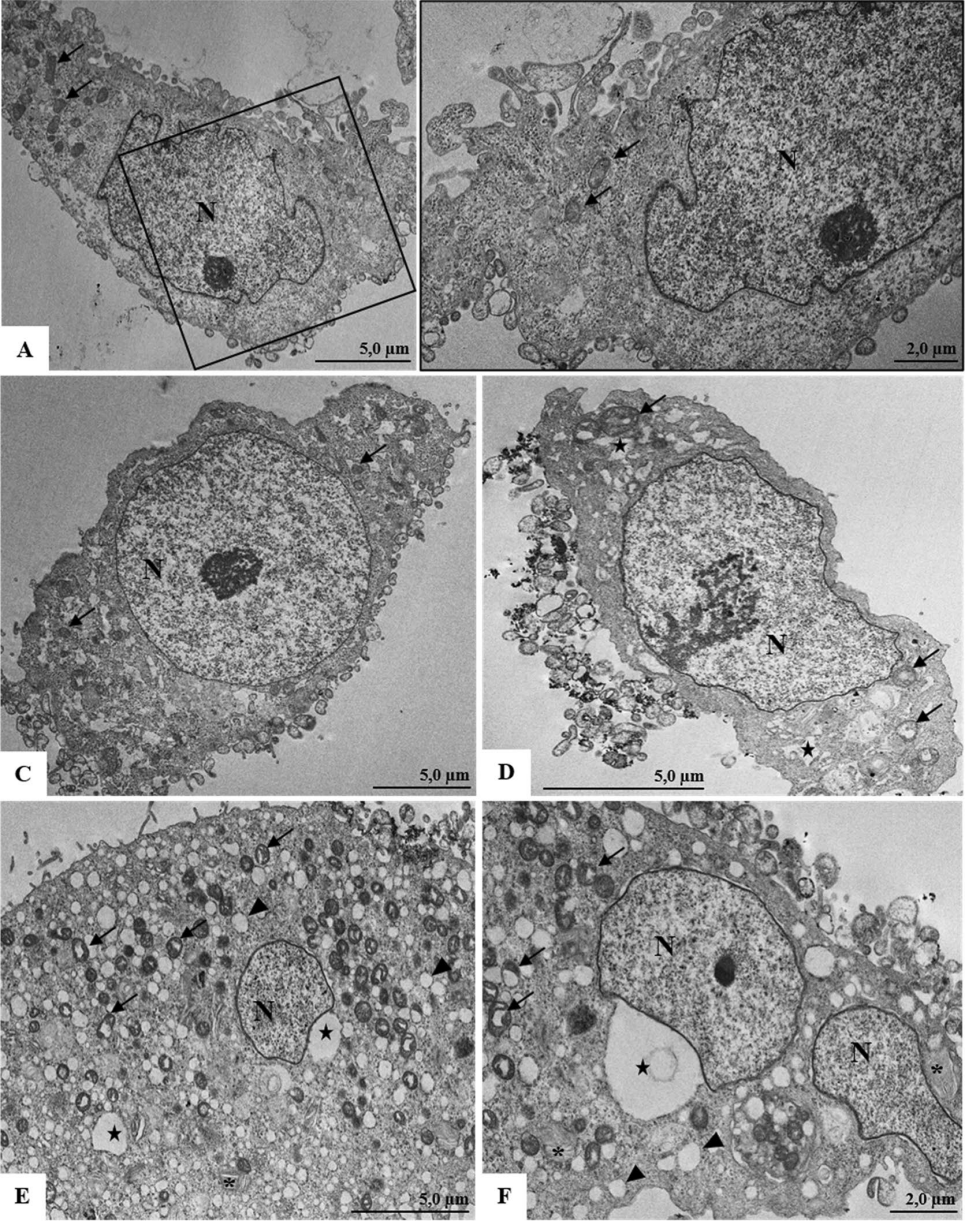
Ultrastructural analyses of Vero-E6 cells untreated and treated with CS6.6/α-Ag_2_WO_4_ composite (**A**–**C**) Untreated cells analyzed with 24 h of cultivation; no morphological alterations were observed; mitochondria (arrow). (**D**–**F**) Cells analyzed 24 h after CS6.6/α-Ag_2_WO_4_ composite treatment presenting vesicles (arrowheads), vacuoles (star), numerous myelin figures (concentric membrane arrays) (*), alterations of mitochondria (arrow). Nucleus (N).

In ultrastructural analyses of untreated Vero-E6 cells and analyzed after 24 h of cultivation, no morphological alterations were observed (Fig. 6A–C). In cells analyzed 24 h after CS6.6/α-Ag_2_WO_4_ treatment, several changes were observed in the cytoplasm, such as proliferation of vesicles, vacuoles, numerous structures with concentric membranes (myelin figures) and changes in mitochondria (Fig. 6D–F), which are indicative of cellular stress.

#### Vero-E6 cells analyzed 1 h after infection with SARS-CoV-2 and treated with PBS, CS6.6 and CS6.6/α-Ag_2_WO_4_ composite

**Figure 7:**
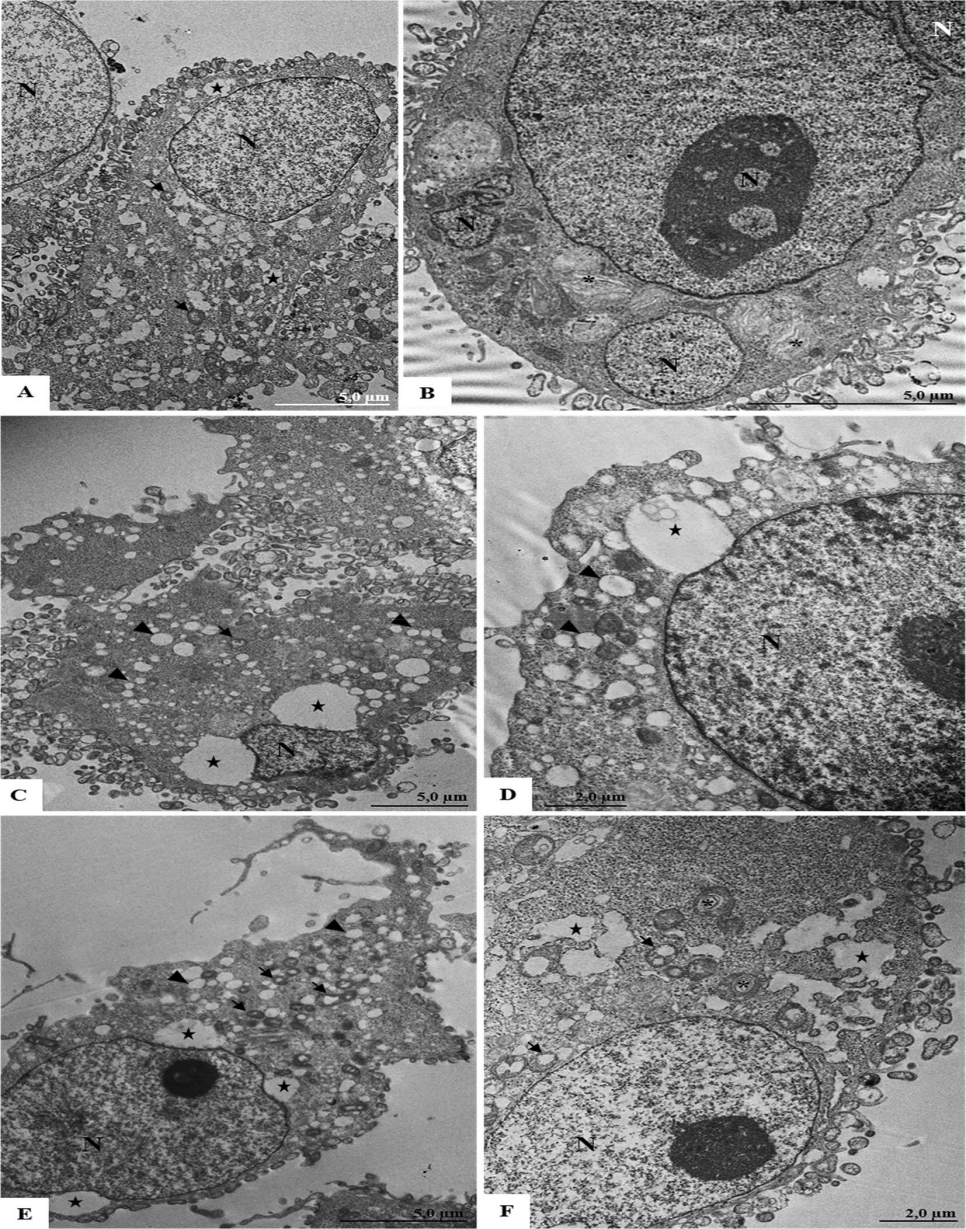
Ultrastructural analyses of Vero-E6 cells 1 h post infection with SARS-CoV-2 treated with PBS, CS6.6 or CS6.6/α-Ag_2_WO_4_ composite. (**A**, **B**) Cells infected with SARS-CoV-2 treated with PBS presenting vacuoles (star), numerous myelin figures (concentric membrane arrays) (*), alterations of mitochondria (arrow) and syncytium (**B**). (**C**, **D**) Cells infected with SARS-CoV-2 treated with CS. Vacuoles (star) and vesicles (arrowheads) was observed. (**E**, **F**) Cells infected with SARS-CoV-2 treated with CS6.6/α-Ag_2_WO_4_ composite presenting vacuoles (star), vesicles (arrowheads), numerous myelin figures (concentric membrane arrays) (*) and alterations of mitochondria (arrow). Nucleus (N).

Cells analyzed 1 h after infection with SARS-CoV-2 and treated with PBS presented morphological alterations in cytoplasm, such as numerous vacuoles, myelin figures and mitochondria alterations. The formation of syncytia (a large cell-like structure formed by joining two or more cells) was also noted (Fig. 7A,B). In cells infected with SARS-CoV-2 and treated with CS6.6, vacuoles and proliferation of vesicles were observed (Fig. 7C,D). A greater number of ultrastructural alterations was found in cells infected with SARS-CoV-2 and treated with the CS6.6/α-Ag_2_WO_4_ composite. The alterations most commonly observed in this case were vacuoles, proliferation of vesicles, numerous myelin figures and mitochondria alterations (Fig. 7E,F). Regardless of the treatments that the viral samples received (PBS, CS6.6 or CS6.6/α-Ag_2_WO_4_ composite), SARS-CoV-2 particles were not detected in the cells within 1 h of infection.

#### Vero-E6 cells analyzed 24 h after infection with SARS-CoV-2 and treated with PBS, CS6.6 and CS6.6/α-Ag_2_WO_4_ composite

**Figure 8:**
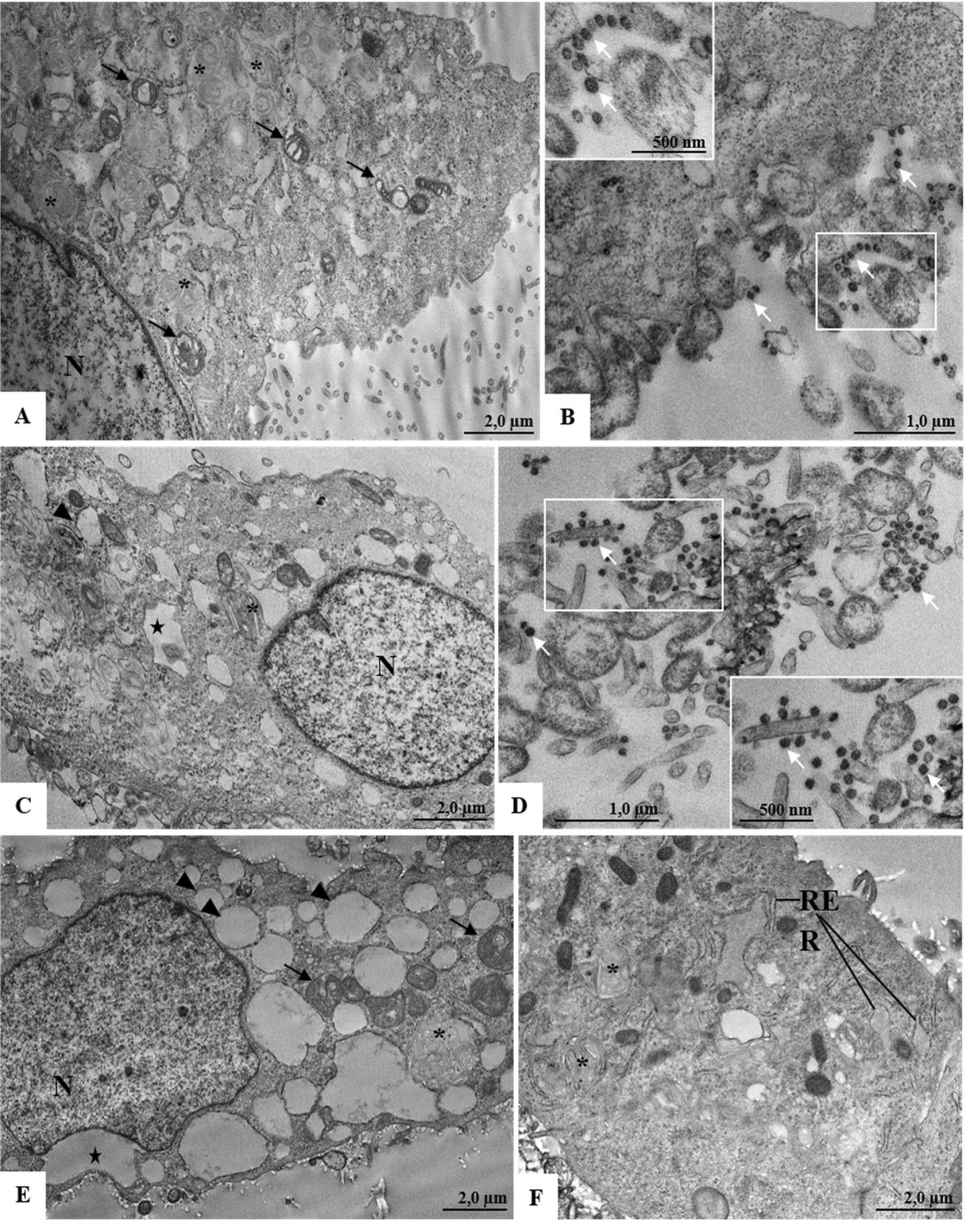
Ultrastructural analyses of Vero-E6 cells 24 h post infection with SARS-CoV-2 treated with PBS, CS6.6 or CS6.6/α-Ag_2_WO_4_ composite. (**A**, **B**) Cells infected with SARS-CoV-2 treated with PBS presenting numerous myelin figures (concentric membrane arrays) (*), alterations of mitochondria (black arrow) and virus particles (white arrow). (**C**, **D**) Cells infected with SARS-CoV-2 treated with CS. Vacuoles (star), vesicles (arrowheads), numerous myelin figures (concentric membrane arrays) (*) and virus particles (white arrow) was observed. (**E**, **F**) Cells infected with SARS-CoV-2 treated with CS6.6/α-Ag_2_WO_4_ composite presenting vacuoles (star), vesicles (arrowheads), numerous myelin figures (concentric membrane arrays) (*) and rough endoplasmic reticulum (RER) thickening. Nucleus (N).

The main morphological changes observed in cells infected with SARS-CoV-2 and treated with PBS were numerous myelin figure and mitochondria alterations (Fig. 8A), besides thickening of the rough endoplasmic reticulum (data not shown). In addition, SARS-CoV-2 particles attached to the plasmatic membrane and in the cytoplasmic vesicle lumen were also observed (Fig. 8B). Cells infected with SARS-CoV-2 and treated with CS6.6 presented in their cytoplasm vacuoles, vesicles and numerous myelin figures (Fig. 8C). Virus particles were found attached to the plasmatic membrane projections (filopodia) (Fig. 8D). In cells infected with SARS-CoV-2 and treated with the CS6.6/α-Ag_2_WO_4_ composite, vacuoles, vesicles, numerous myelin figures and rough endoplasmic reticulum thickening (Fig. 8E,F) were the main morphological changes observed. No SARS-CoV-2 particles were observed.

In summary, ultrastructural analyses of Vero-E6 cells 24 h after CS6.6/α-Ag_2_WO_4_ composite treatment presented morphological alterations, indicating cytotoxicity. No SARS-CoV-2 particles were detected in the monolayer analyzed 1 h after infection with SARS-CoV-2 and treatment with PBS, CS6.6 and CS6.6/α-Ag_2_WO_4_ composite. On the other hand, cells infected with SARS-CoV-2 and treated with PBS presented syncytia formation, which is a cytopathic effect observed in cell cultures and tissues in individuals infected with SARS‐CoV‐1, MERS‐CoV, or SARS‐CoV‐2^72–83^. It is believed that the non-detection is due to the small number of particles that may be associated with short infection time. Additionally, in this period of infection the particles would be in the process of assembly.

Different from the observations after 1 h of infection, Vero-E6 cells analyzed 24 h after infection with SARS-CoV-2 and treated with PBS or CS6.6 presented SARS-CoV-2 particles attached to the cell surface and inside cell vesicles, proving that both PBS and CS6.6 were not capable of inhibiting virus synthesis. Finally, although several morphological alterations were observed in cells infected with SARS-CoV-2 and treated with the CS6.6/α-Ag_2_WO_4_ composite, no viral particles were found, which may be attributed to the virucidal action.

### Proposed mechanism for the biocidal activity

**Figure 9:**
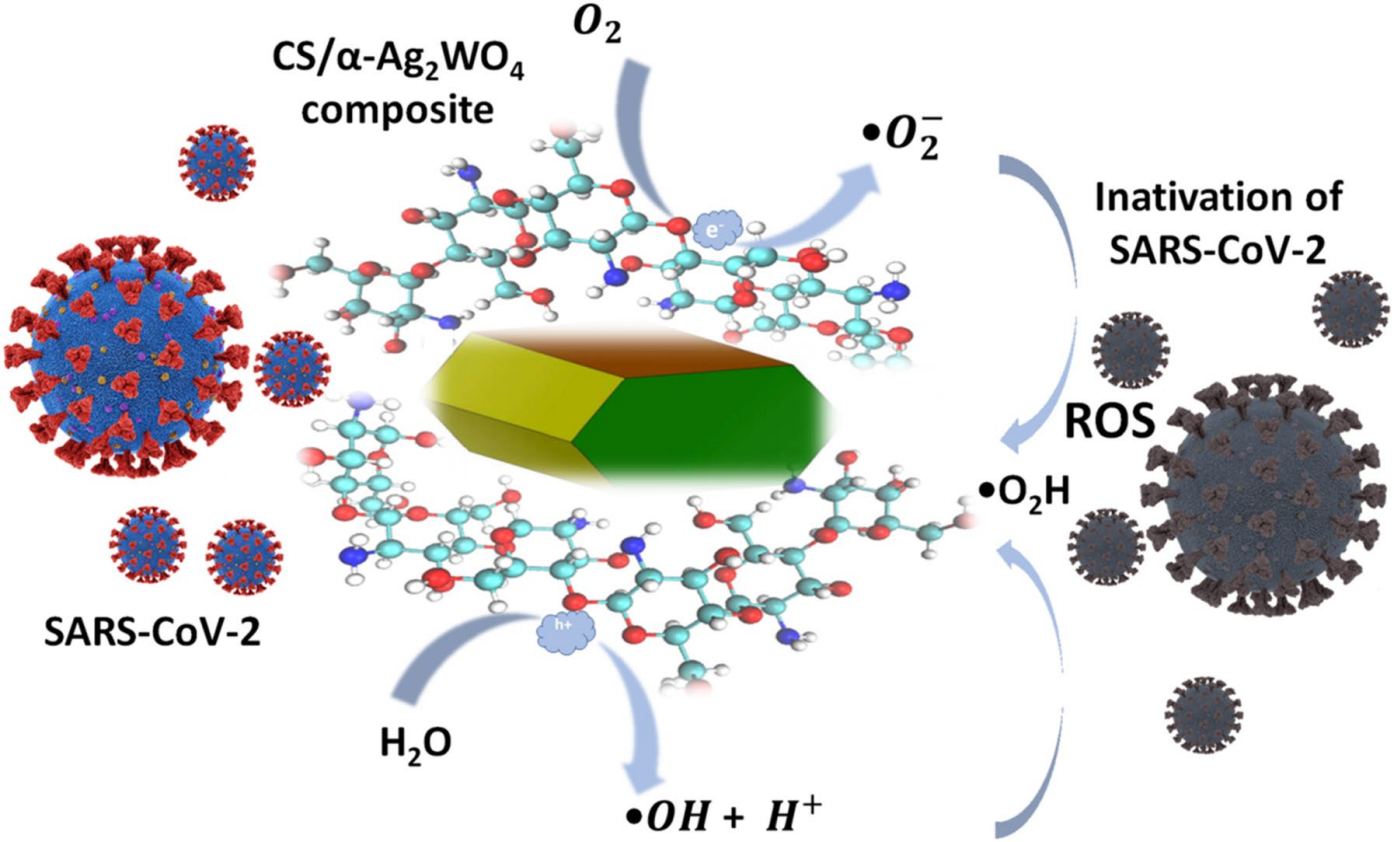
Schematic representation of biocidal activity of CS/α-Ag_2_WO_4_ composite due to ROS generation.

The proposed biocidal mechanism of the CS/α-Ag_2_WO_4_ composites is summarized in Fig. 9. It is possible to note that CS presents strong affinity with metal ions as a result of the presence of –OH and –NH_2_ groups, which can reduce Ag^+^ ions to AgNPs^55^. Thus, due to the consequent interaction with the fs laser irradiation, the AgNPs are formed in the system. According to Jena et al.^68^, the presence of AgNPs lead to the formation of reactive oxygen species (ROS), causing DNA damage, and consequently producing changes in its conformation. As a result, the aforementioned composites absorb the incident photons, and the electrons $(e^{ - } )$ in the VB are excited to the CB; at the same time, holes (*h*•) are generated in the VB. Moreover, the presence of AgNPs increases the population of $e^{ - }$ in the CB of the semiconductor due to their surface plasmon resonance (SPR) effect, causing an accumulation of positive vacancies in the VB. The strong SPR effect of AgNPs in these composite systems helps to effectively transfer the photogenerated carriers, thereby facilitating the charge separation at the composite interface, drastically improving the biocidal activity of the composite compared to that of the counterparts. Therefore, the enhanced presence of *h*• in the VB causes a strong interaction with the H_2_O molecule, leading to the formation of •OH and H^+^. Simultaneously, the O_2_ molecule is converted into •O_2_^−^ in the CB of the semiconductor due to the reaction with $e^{ - }$. In addition, the protonation of •O_2_^−^ renders the •O_2_H radical. It is reported that the oxidative stress is caused by imbalances in the production and elimination of ROS, resulting in biocidal activity^18^. It also prevents the vital function of the cell, affecting the viability, proliferation and redox status of various cell types^41^, thus destabilizing cell wall and membrane, and consequently leading to, cell death^27^.

## Conclusions and outlook

The rapidly spreading outbreak of COVID-19 has challenged the world’s healthcare sector over the last year. Thus, it has become crucial to trap and eradicate SARS-CoV-2 by using new materials. In this work, we reported the synthesis of chitosan/α-Ag_2_WO_4_ composites generated by femtosecond laser irradiation. This material is very efficient to eliminate bacteria (*Escherichia coli*, *Methicillin-susceptible Staphylococcus aureus*, and the yeast strain *Candida albicans*) and SARS-CoV-2 by contact. This study offers a general strategy to construct biocide materials. The biomimetic function of CS/α-Ag_2_WO_4_ composites in defeating COVID-19 transmission is promising. However, further studies are still necessary for developing new technologies based on the functionalization of this composite applied on protective materials and communal objects (e.g., mask, door handles, elevator buttons, gas pumps, and handrails) to reduce both disease transmission and fear of touching objects.

## Experimental section

### Synthesis of α-Ag_2_WO_4_ microcrystal

The α-Ag_2_WO_4_ microcrystal was prepared by the coprecipitation (CP) method as previously described^46^. The procedure is described as follows: 2 × 10^−3^ mol of silver(I) nitrate (AgNO_3_; 99.8% purity, Sigma-Aldrich) was dissolved in 50 mL of deionized water at 80 °C under magnetic stirring and then this solution was added in 50 ml of 1 × 10^−3^ mol of sodium tungstate (VI) dehydrated (Na_2_WO_4_·2H_2_O; 99.5% purity, Sigma-Aldrich) previously dissolved at the same temperature. The suspension formed remained under constant magnetic stirring for 30 min. The resulting powders were washed several times with deionized water and dried in an oven at 70 °C.

### Preparation of the chitosan (CS)

The experimental procedure employed for the synthesis of CS was obtained according to Ref.^54,84^. Briefly, 0.33, 0.66 and 0.99 g of the CS (75–85% deacetylated, Sigma-Aldrich) were dissolved in 100 ml of 0.5% (v/v) acetic acid (AA) under constant stirring for 24 h at 25 °C, getting 3.3, 6.6 and 9.9 g/L concentration of CS solution.

### Femtosecond laser irradiation of CS/α-Ag_2_WO_4_ composites

The CS/α-Ag_2_WO_4_ composite was obtained according to Ferreira et al. and Ancona et al.^61,62^. Briefly, 400 mg of α-Ag_2_WO_4_ microcrystal was dispersed in 15 mL of CS solution, under constant stirring to complete homogenized the dispersion, getting 26.670 µg/mL concentration. To perform the femtosecond laser irradiation, a Femtosecond Ti: sapphire laser (Femtopower Compact Pro, Femto Lasers) of 30 fs full width at half maximum (FWHM) pulse duration emitting at the central wavelength of 800 nm, with a repetition rate of 1 kHz was employed. From the different setups usually used for laser irradiation, as the batch and a flow jet configuration^85^, the batch configuration was selected due to the simplicity of the technique and the lack of a requisite for a pumping device. In the batch processing the dispersed CS/α-Ag_2_WO_4_ composites was contained in a glass cell, the laser beam was focused perpendicular to the surface and during irradiation a magnetic stirrer was used to expedite the movement and prevent gravitational settling. The setup is shown in Fig. S1. To find the right parameters of irradiation, different parameters were tested with smaller sample volumes. Finally, a laser beam of 6 mm in diameter, 1/e^2^ criteria, mean power of 150 mW and irradiation during 2 h were found to be the optimum parameters to complete the CS/α-Ag_2_WO_4_ processing.

### Structural characterization of the α-Ag_2_WO_4_ microcrystals and CS/α-Ag_2_WO_4_ composites

The α-Ag_2_WO_4_ microcrystals and CS/α-Ag_2_WO_4_ composites were structurally characterized by XRD patterns using a D/Max-2000PC diffractometer Rigaku (Japan) with Cu Ka radiation (λ = 1.5406 Å) in the 2θ range from 10° to 110° in the normal routine with a scanning velocity of 2°/min. Fourier-transform infrared (FTIR) spectra were obtained on a Bruker spectrometer (Vertex 70) equipped with a detector of triglycine sulfate doped with L-alanine and deuterium (DLaTGS). The spectra were collected in the spectral range from 50 to 4000 cm^−1^ with a nominal resolution of 4 cm^-1^. The shapes and sizes of the α-Ag_2_WO_4_ microcrystals and α-Ag_2_WO_4_/CS composites were observed with an FE-SEM Inspect F50 (FEI Company, Hillsboro, OR) operated at 5 kV. The bifunctional nanoparticles were characterized by High-resolution *transmission electron microscopy* (HRTEM) using a JEM 2100F TEM/STEM micro-scope operating at 200 kV.

### Antimicrobial assays

Previous to each assay, the CS/α-Ag_2_WO_4_ composites irradiated solutions at 26.670 µg/mL were diluted in RPMI-1640 medium supplemented (with 25 mM HEPES (Sigma) and 2 g/L sodium bicarbonate (Synth) pH 7.0 ± 0.2)^86^ at final concentration of 2.000 µg/mL. A serial dilution 1:2 was made in flat bottom 96-well plates. A concentration range of 1.000–0.49 µg/mL was obtained after adding a 100 µL volume of inoculum to each well.

### Microorganisms and culture conditions

The antimicrobial activity of CS/α-Ag_2_WO_4_ composites irradiated by fs laser were evaluated by experiments using two strains of bacteria (Gram-positive and Gram-negative) and a strain of fungi. The bacteria strains of *Escherichia coli* (ATCC 25,922) and *Methicillin-susceptible Staphylococcus aureus* (MSSA—ATCC 25,923), and the yeast strain *Candida albicans* (ATCC 90,028) were maintained frozen at − 80 °C until its use. *E. coli* and *S. aureus* were plated on Brain Heart Infusion (BHI) agar, and *C. albicans* on Sabouraud Dextrose Agar (SDA) for 24 h previously to pre-inoculum. It was made a prior overnight pre-inoculum in Tryptic Soy Broth (TSB) to bacterial strains, and Yeast Nitrogen Base medium (YNB) with 100 mM glucose to yeast strain. The inoculums were prepared diluting the pre-inoculum in 1:30 to *E. coli*, 1:20 to *S. aureus* and *C. albicans*, in their respective media. They were incubated at 37 °C under static conditions until microorganisms reached the mid-log phase (according to growth curve pre-established). The inoculums were centrifuged (4000 rpm, 4 °C, 10 min) and the pellet was washed twice with phosphate-buffered saline (PBS, pH 7.0), and resuspended in RPMI-1640 at initial volume. All three microorganisms were diluted in RPMI-1640 to obtain the final concentration 1 × 10^3^ to 5 × 10^3^ CFU/mL for *C. albicans*, and 5 × 10^5^ CFU/mL for *S. aureus* and *E. coli,* according to the protocols standardized by CLSI (M7-A6, 2003; M27-A3, 2008).

### Antimicrobial activity of CS/α-Ag_2_WO_4_ composites

The minimal inhibitory concentration (MIC) and the minimal bactericidal/fungicidal concentration (MBC/MFC) against planktonic cells were determined using a broth microdilution method, as described by the Clinical and Laboratory Standards Institute (CLSI), documents M27-A3 and M7-A6. MIC and MBC/MFC were determined by incubating of *S. aureus*, *E. coli* and *C. albicans* directly into a 96-well plate containing CS/α-Ag_2_WO_4_ composites irradiated by fs laser, in final concentration of 1000–0.49 µg/mL, for 24 h at 37 °C. The MIC was determined by visual inspection, where it was considered the lower concentration without visual growth. The MBC/MFC values were determined by cell recovery in an agar culture medium. For that, the MIC and 5 concentrations higher than the MIC were submitted to tenfold serial dilution in PBS. Each dilution was plated by microtip methodology on petri dishes with specific agar medium, and the plates were incubated at 37 °C, overnight. Counting the number of colonies was carried out in the lowest possible dilution. The data were converted to Log_10_ (UFC/mL) and converted in inhibition index related to control without treatment. The literature describes MFC/MBC as the minimum concentration of the antimicrobial agent capable of killing 99.9% of the number of colonies (CFU/mL) or reducing 3 units of Log_10_ in relation to the untreated control^87–90^. As control were used microorganisms in standard culture (CT), and the vehicles (CS and AA) at the same concentrations of experimental groups (to evaluate their interference in the cell viability). This experiment was performed in quadruplicate and in two different occasions (n = 8).

### Cytotoxicity assays in NOK-si cell

#### Cell line and culture conditions

To evaluate the cytotoxicity of CS/α-Ag_2_WO_4_ composites irradiated by fs laser, the NOK-si cell line (normal oral keratinocyte spontaneous immortalized), kindly provided by Professor Carlos Rossa Junior (Department of Diagnosis and Surgery, Faculty of Dentistry of Araraquara-UNESP)^91^ was used. NOK-si was grown in Dulbecco's Modified Eagle's Medium (DMEM) medium with 4.5 g/L glucose (Sigma-Aldrich), supplemented with 2.0 mM of L-glutamine (Lonza), 1% of antibiotic/antimycotic solution (Sigma-Aldrich) and 10% fetal bovine serum (FBS; Gibco) in 75 cm^2^ bottle (Kasvi) at 37 °C, 5% CO_2_. After cells reach 80% confluence, they were washed with PBS, detached from the apparatus with trypsin solution (0.05% trypsin /0.53 mmol/L EDTA) (Sigma-Aldrich) and centrifuged at 400 × *g* for 5 min. The cell pellet was resuspended in culture medium, and viable cells index was verified by trypan blue methodology (1:1/v:v) (Sigma-Aldrich) using the automatic Countess II FL counter (LifeTechnologies). Calculations were performed to plate 5 × 10^3^ cells/well in a 96-well plate. The plates were incubated at 37 °C, 5% CO_2_. After cells reach 60% confluence, the cells were washed once with PBS and treated with 200 µL of CS/α-Ag_2_WO_4_ composites irradiated by fs laser (serial dilution in RPMI-1640 supplemented from 31.25 to 0.49 µg/mL). The plates were incubated for 24, 48 and 72 h at 37 °C, 5% CO_2_. The data obtained for MTT and Alamar Blue assay (followed described) were converted to viability index in relation to control without treatment. The experimental controls were wells with cell monolayer without treatment (CT), culture medium without cell monolayer (sterility test), and vehicles (CS and AA) to evaluate their interference in the cell viability. From the volume of vehicle used in the first concentration of the experimental solution, 6 serial 1:2 dilutions were made in culture medium (C1–C7), with C1 being the initial one, and C7 with the highest dilution. The tests were performed in quadruplicate and on three independent occasions (n = 12).

#### MTT assay

This assay assesses the rate of viable cells by mitochondrial activity (vitality assay) by quantifying tetrazolium salt reduction to formazan crystals, which occurs mainly by succinic dehydrogenase enzymes in mitochondrial fraction. After the incubation time (24, 48 and 72 h), the supernatant was removed and it was added 100 µL/well of MTT solution (3-(4,5-dimethyl-2-thiazolyl)-2,5-diphenyl-2*H*-tetrazolium bromide; 1.2 mg/mL; Sigma-Aldrich) in RPMI-1640 medium without phenol red (Sigma-Aldrich). The plate was incubated at 37 °C, 5% CO_2_. After 4 h, the supernatant was discarded and 100 µL/well of isopropanol (Synth) were added to solubilize the formazan crystals. Each well was homogenized, and the plate was submitted to analysis in a spectrophotometer at 540 nm. This protocol was performed at 24, 48 and 72 h of incubation.

#### Alamar blue assay

In this assay, the viability rate was quantified by the metabolic activity of viable cells. The reagent has the compound resazurin (7-hydroxy-3H-phenoxazin-3-one-10-oxide/LifeTechnologies) which is a non-fluorescent blue dye. This is reduced by reductase enzymes, present in the cytosol and mitochondria, to a highly fluorescent pink dye, resorufin. After 20 h of the cells being challenged with the CS/α-Ag_2_WO_4_ composites, 20 µL of the alamar blue solution was added to each well. The plate was incubated for 4 h at 37 °C, 5% CO_2_ and the analysis in a spectrophotometer at 540 nm (600 nm reference filter) for 24 h was performed. The same plate was incubated at 37 °C, 5% CO_2_ to perform the analysis of times 48 and 72 h, since resazurin is a non-invasive and stable probe.

#### Statistical analysis for cytotoxicity assay

Shapiro–Wilk and Levene’s test were performed to test data distribution and homogeneity. Based on normal and heteroskedastic distribution, statistical comparisons were performed by one-way analysis of variance (ANOVA) with Welch correction, followed by Games Howell Post Hoc, using BM SPSS Statistics program (version 23). All data are plotted as the mean + standard deviation (SD) and p < 0.05 was considered statistically significant.

### Antiviral assays

#### Virus inactivation assay

Firstly, with the aim of neutralizing SARS-CoV-2 (GISAID EPI ISL #414,045, SisGen ACCF49F), 3.0 × 10^4^ TCID50 of the virus were incubated for 10 min with CS6.6/α-Ag_2_WO_4_ (4.0 µg/mL) composite at 37 °C using a protocol that was previously described^92^ with some adaptations. The CS6.6 solution, in the same concentration of CS6.6/α-Ag_2_WO_4_ composite, and PBS were used as control groups. After this step, each virus inactivated solution was divided equally in 2 parts to analyze virucidal effect 1 and 24 h post infection (hpi) in cell culture. This assay was repeated 3 times. All procedures related to virus culture were handled at biosafety level 3 (BSL3) multiuser facilities, according to World Health Organization (WHO) guidelines.

#### Evaluation of virucidal effect

To study the capacity of CS6.6/α-Ag_2_WO_4_ inactivate SARS-CoV-2 activity, Vero-E6 cells monolayers were incubated with viral inactivated solutions and the virus in supernatants were analyzed 1 and 24 hpi. For this purpose, Vero-E6 cells (1.5 × 10^6^) previously seeded, maintained in Dulbecco’s Modified Eagle’s Medium (DMEM, Gibco) supplemented with 10% Fetal Bovine Serum (FBS, Gibco) in cell culture 24 cm^2^ flasks, were incubated with each viral inactivated solution in multiplicity of infection (MOI) 0.01 for 1 h at 37 °C and 5% CO_2_ . Then, the supernatants were harvested and the cell monolayers washed twice with PBS and physically removed from the flasks for processing for transmission electron microscopy (TEM) analysis, or incubated with DMEM/HEPES/2% FBS for 24 h, before harvesting the supernatant and preparation to TEM. The supernatants were stored at − 70 °C for posterior virus titration and RNA quantification by Plaque Forming Units (PFU/mL) and qRT-PCR (number of copies/mL), respectively^93,94^.

### Virus titration and RNA quantification

For PFU assay, monolayers of Vero-E6 cells (10^5^ cells/well) were seeded into 24-well culture plates (flat bottom) and grown for 24 h at 37 °C in 5% CO_2_. These cells were inoculated with 300 µL of infected cells supernatants dilutions (10^−1^ to 10^−4^). After 1 h at 37 °C in 5% CO_2_, the medium was changed to 500 µL of a solution containing DMEM-High glucose 1X, 1.8% carboxymethylcellulose and 2% FBS. 72 h post infection, cytopathic effects (CPE) were observed on optical microscope and cells fixed with 10% formalin. After 3 h, this solution was removed and monolayers stained with 0.04% crystal violet for Plaque Forming Units counting^93^. The molecular detection of viral RNA levels was performed as described before^94^. Primers, probes, and cycling conditions recommended by the Centers for Disease Control and Prevention (CDC) protocol were used to detect the SARS-CoV-2 envelope gene (E)^95^. Cell supernatants were used for viral RNA quantification by real time qRT-PCR and were expressed in number of copies of virus RNA per mL. Concurrently to viral RNA amplification, standard curves were plotted with different numbers of copies per cycle threshold (Ct). The standard curve method was used in comparison with the viral gene to obtain the relative quantification of the viral RNA in supernatants^96^.

### Transmission electron microscopy (TEM)

For TEM analyses the Vero-E6 cells suspensions were fixed in 2.5% glutaraldehyde in sodium cacodilate buffer (0.2 M, pH 7.2), post-fixed in 1% buffered osmium tetroxide, dehydrated in acetone, embedded in epoxy resin and polymerised at 60 °C over the course of three days^81,82^. Ultrathin sections (50–70 nm) were obtained from the resin blocks. The sections were picked up using copper grids, stained with uranyl acetate and lead citrate^83^, and observed using Hitachi HT 7800 transmission electron microscope.

### Statistical analysis for antiviral assays

The data were analyzed using Past program^97^. The one-way ANOVA with Tukey post-test was performed to determine the significance between the different experimental groups (CS6.6/α-Ag2WO4, CS6.6 and PBS). The graphics were plotted by the OriginLab Pro 2021 program. Results are presented as the mean of 3 independent experiments ± standard deviation (SD) with a confidence interval of 95%, and significant *p* values were represented as ^d^ for < 0.005 and ^e^ for < 0.05.

## Supporting Information

Detailed information about the XRD, FTIR, FE-SEM, TEM, and cytotoxicity discussion are described in the Electronic Supporting Information.

## Supplementary Information

Supplementary Information.
