# Supplementary material for: Inactivation of SARS-CoV-2 by a chitosan/α-Ag2WO4 composite generated by femtosecond laser irradiation
Source: Sci Rep. 2022 May 17;12:8118. doi: 10.1038/s41598-022-11902-5 (PMC9114143; doi:10.1038/s41598-022-11902-5)
Supplement: Supplementary file 1 — Supplementary Information. [file 41598_2022_11902_MOESM1_ESM.docx]

Supporting Information (SI) for:

**Inactivation of SARS-CoV-2 by a Novel Chitosan/α-Ag_2_WO_4_ Composite Generated by Femtosecond Laser Irradiation**

Paula Fabiana Santos Pereira,*^1,8^* Ana Carolina Alves de Paula e Silva,*^2^* Bruna Natália Alves da Silva Pimentel,*^2^* Ivo Mateus Pinatti,*^3,8^* Alexandre Zirpoli Simões,*^3^* Carlos Eduardo Vergani,*^2^* Débora Ferreira Barreto-Vieira,*^4^* Marcos Alexandre Nunes da Silva,*^4^* Milene Dias Miranda,*^5^* Maria Eduarda Santos Monteiro,*^5^* Amanda Tucci,*^5^* Carlos Doñate-Buendía*,^6,7^* Gladys Mínguez-Vega,*^6^* Juan Andrés,*^8^* and Elson Longo *^1^* *

*^1^* CDMF, LIEC, Department of Chemistry, Federal University of São Carlos (UFSCar), P.O. Box 676, 13565-905 São Carlos, SP, Brazil.

*^2^* Department of Dental Materials and Prosthodontics, São Paulo State University (UNESP), School of Dentistry, Araraquara, São Paulo, Brazil. 1680 Humaitá Street. 14801-903.

*^3^* Faculty of Engineering of Guaratinguetá, São Paulo State University (UNESP), 12516-410, Guaratinguetá, SP, Brazil.

*^4^* Laboratory of Viral Morphology and Morphogenesis, Oswaldo Cruz Institute, Fiocruz, Avenida Brasil, Rio de Janeiro, Brazil.

^5^ Laboratory of Respiratory Viruses and Measles, Oswaldo Cruz Institute, Fiocruz, Avenida Brasil, Rio de Janeiro, Brazil

*^6^* GROC UJI, Institute of New Imaging Technologies, Universitat Jaume I, Avda. Sos Baynat sn, Castellón de la Plana, 12071, Spain.

*^7^* Materials Science and Additive Manufacturing, University of Wuppertal, Gaußstr. 20, 42119 Wuppertal, Germany

*^8^* Department of Physical and Analytical Chemistry, University Jaume I (UJI), Castelló 12071, Spain.

* elson.liec@gmail.com

# Experimental section


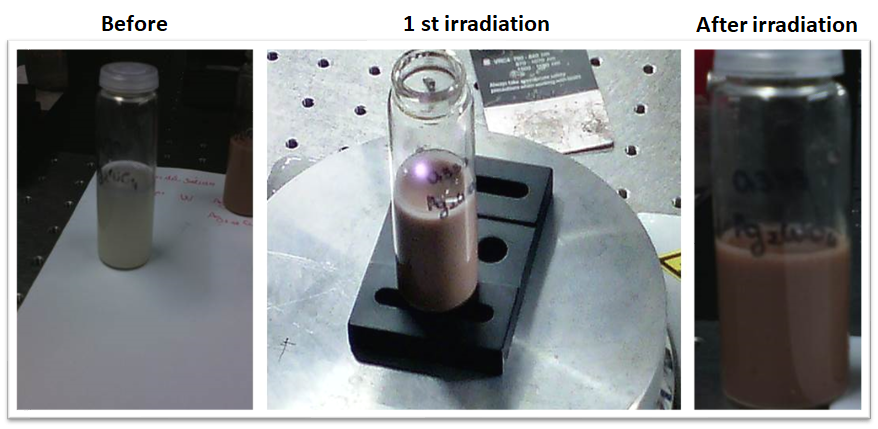


**Figure** **S1.** From left to right: Before, during, and after 2 hours of irradiation of CS/α-Ag_2_WO_4_ composites.

# Results and Discussion

# Structural analysis


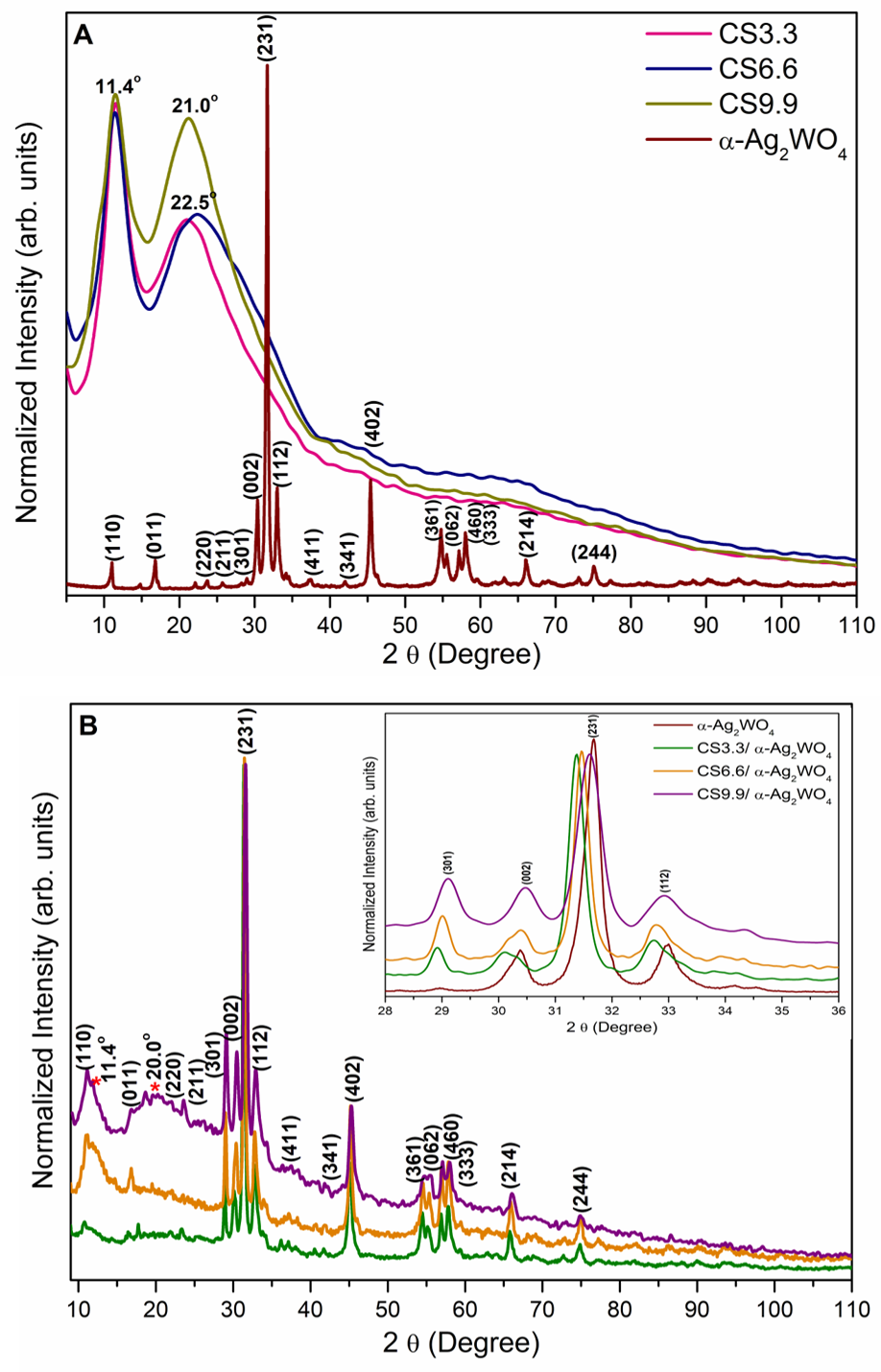


Figure S2. XRD patterns of (A) α-Ag_2_WO_4_ microcrystal, and CS-3.3, CS-6.6 and CS-9.9 polymers, and (B) CS-3.3/α-Ag_2_WO_4_, CS-6.6/α-Ag_2_WO_4_ and CS-9.9/α-Ag_2_WO_4_ composites irradiated by fs laser.

The FTIR spectra analysis of the CS3.3, CS6.6 and CS9.9 polymers were observed all the bands characteristics of the CS polymer: the peaks at 3359 and 3258 cm^-1^, associated to the stretching vibrations are attributed to hydroxyl (-OH) and amine (-NH_2_) groups, respectively, the symmetric or asymmetric stretching of CH_2_ group at 2929, 2875, 1404 and 1324 cm^-1^, belong to pyranose ring ^1-5^. The bands at 1636, 1542 and 1377 cm^-1^ are assigned to stretching band (C=O), bending vibrations NH (from amino group) ^1,2^, and bending band (CH_3_) from amide group, respectively. The bands in the range 1150–1020 cm^-1^ belong to asymmetric vibrations C–O–C from glycosidic group ^1,2^, the band at 1153 cm^-1^ is assigned to asymmetric vibrations C–O–C of glycosidic group and the bands at 1063 and 1022 cm^-1^ are attributed to skeletal stretching =CO, which correspond to bands of polysaccharides ^1-3^. In the range of 2000–1650 cm^-1^ no bands were observed, which are associated to (–C=O) carbonyl group vibration ^6^. According to Klan et. al. ^6^ the absence of these peaks indicates that there was no degradation of CS polymers after fs irradiation. The band at 653 cm^-1^ belong to the bending vibrations of amine groups and the band at 902 cm^-1^ corresponds to pyranose ring (see Fig. ESI-3).


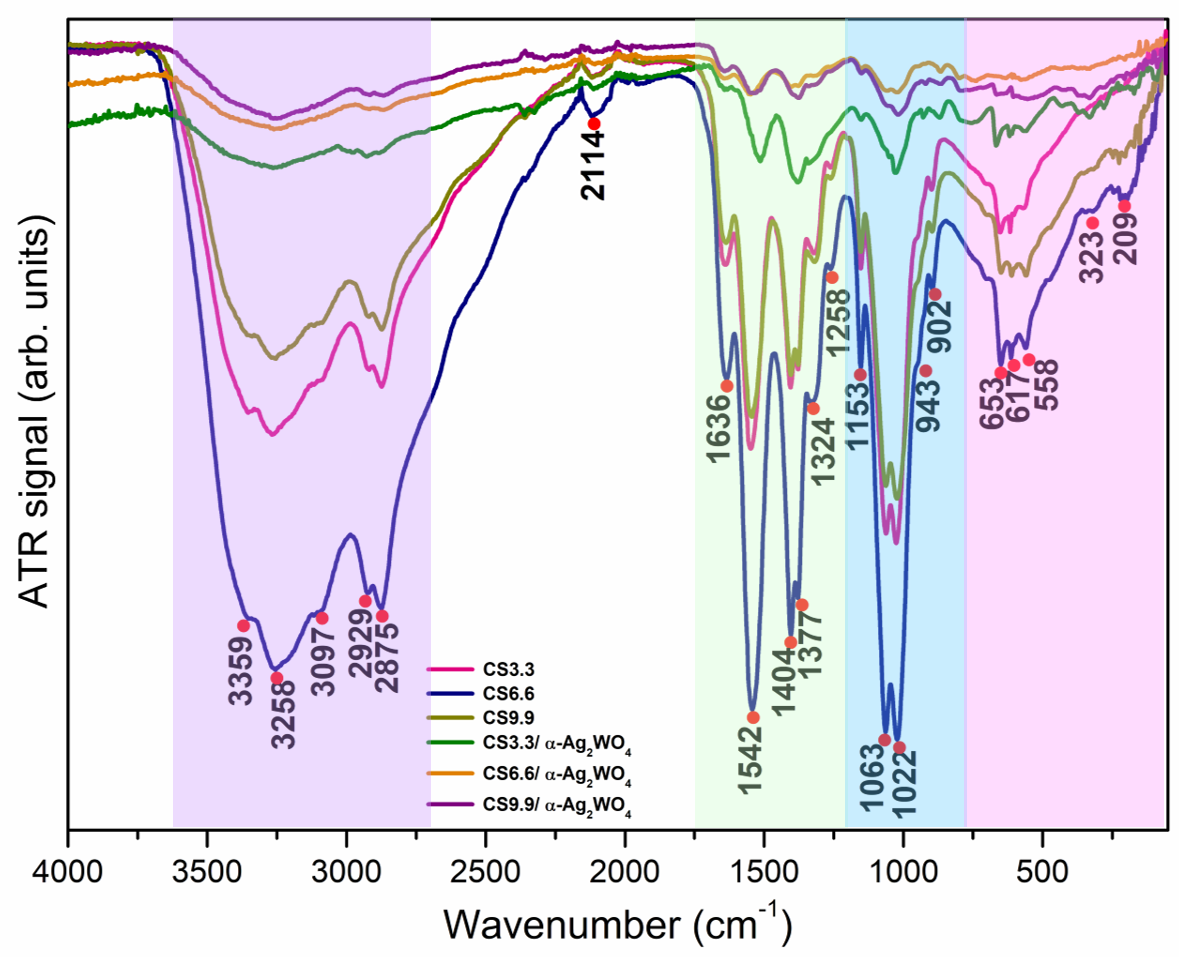


Figure S3. ATR-FTIR spectra of CS3.3, CS6.6 and CS9.9 polymers and CS3.3/α-Ag_2_WO_4_, CS6.6/ α-Ag_2_WO_4_ and CS9.9/ α-Ag_2_WO_4_ composites irradiated by fs laser.

Fragments of FTIR spectra were done in the range 4000–2400 cm^-1^, 2000–1200 cm^-1^, 1200–800 cm^-1^ and 800–50 cm^-1^ (Fig. ESI-4) to study the CS/α-Ag_2_WO_4_ composites irradiated by fs laser and the wavenumber positions are listed in Table ESI-1. These results showed similar behavior, however, a general decrease in absorbance intensity and small shift in the bands was noticed for all the CS/α-Ag_2_WO_4_ composites (see Table ESI-1).

In accordance to Klan et. al. ^6^ the presence of the band at around 900 cm^-1^, which has been shifted to higher wavenumber in CS/α-Ag_2_WO_4_ composites (see Table ESI-1), confirms that the pyranose ring remained preserved after irradiation, and also the presence of the peaks at 1636, 1542 and 653 cm^-1^ corresponding to amine group, which kept on stable during the irradiation in femtosecond laser. The decrease of band at 3359 cm^-1^ assigned to ν(OH) and the small shift at around the 1636 cm^-1^ ν(C=O) and 1542 cm^-1^ δ(NH);ν(CH) confirms the formation of intermolecular hydrogen bond of the OH and amino group of the CS with α-Ag_2_WO_4_ compound ^7,8^ and the decreasing of the intensity of the absorption at around 1404 cm^-1^ confirm these displacements, according to Ferreira et. al. ^5^. Tiwari et. al. ^9^ reported the absorbance stretching band at 1258 cm^-1^ characteristic of the (–C–N) group of CS polymer becomes flattened due to the bonding interaction between the metal, in this case of the α-Ag_2_WO_4_ with the (–C–N) group (see Fig. ESI-4B and Table ESI-1). It is observed different displacement of absorbance peaks of the CS/ α-Ag_2_WO_4_ composites in relation to CS polymers, probably this behavior is the different amount of intermolecular hydrogen bond of the ν(OH) and amino group δ(NH);ν(CH) disponible to bonding.

Several peaks of absorbance arise in the ATR-FTIR spectra of CS/α-Ag_2_WO_4_ composites in the range from 867 to 50 cm^-1^ (see Fig. ESI-4 C and D and Table ESI-1), which are attributed to the distorted O–W–O bonds of the tetrahedral ${[WO}_{4}^{2-}]$ clusters and Ag–O, of the α-Ag_2_WO_4_ compound. In our previous works ^10^ all the absorbance peaks were characterized and discussed. However, the active mode at around 867 cm^-1^, 760–743 cm^-1^, 552–332 cm^-1^ are assigned to asymmetric stretching vibration modes of the W–O–W and O–W–O characteristic of tetragonal ${[WO}_{4}^{2-}]$ clusters (see inset Fig. ESI-4 C). The active mode at around 630–616 cm^-1^ is attributed to bridging oxygen atoms in the W_2_O_2_ asymmetric stretching and the absorption bands at around 664–642 cm^-1^ is related to the vibrations of the O–W–O bonds ^10-15^. Finally, the active modes below to 250 cm^-1^ are characteristics to the Ag–O bonds. All these behaviors prove the success of the obtention of CS/α-Ag_2_WO_4_ composites in different concentrations.

**
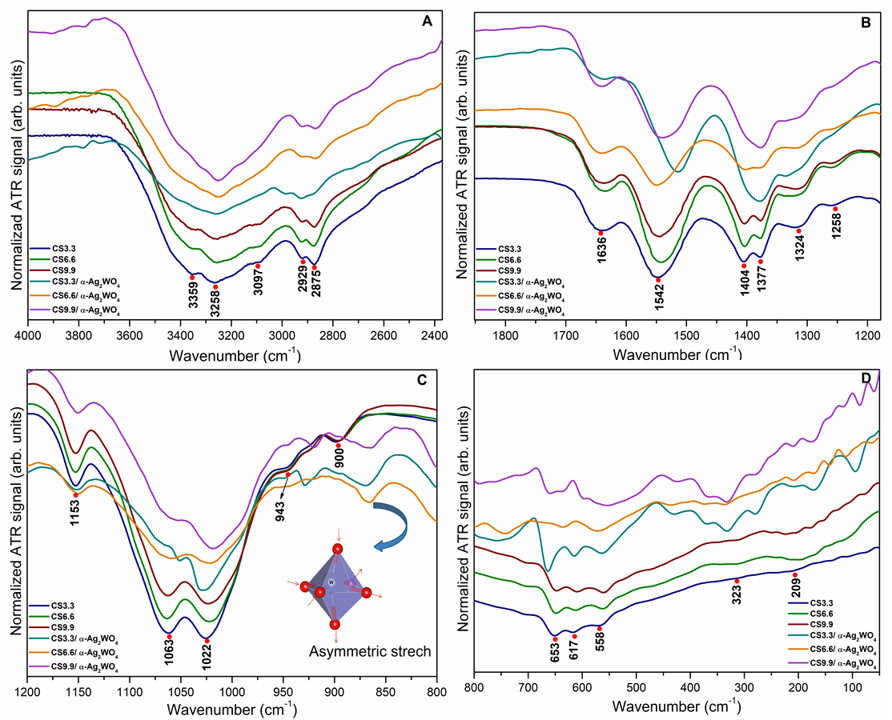
**

Figure S4. ATR-FTIR spectra of CS3.3, CS6.6 and CS9.9 polymers and CS3.3/α-Ag_2_WO_4_, CS6.6/α-Ag_2_WO_4_ and CS9.9/α-Ag_2_WO_4_ composites irradiated by fs laser.


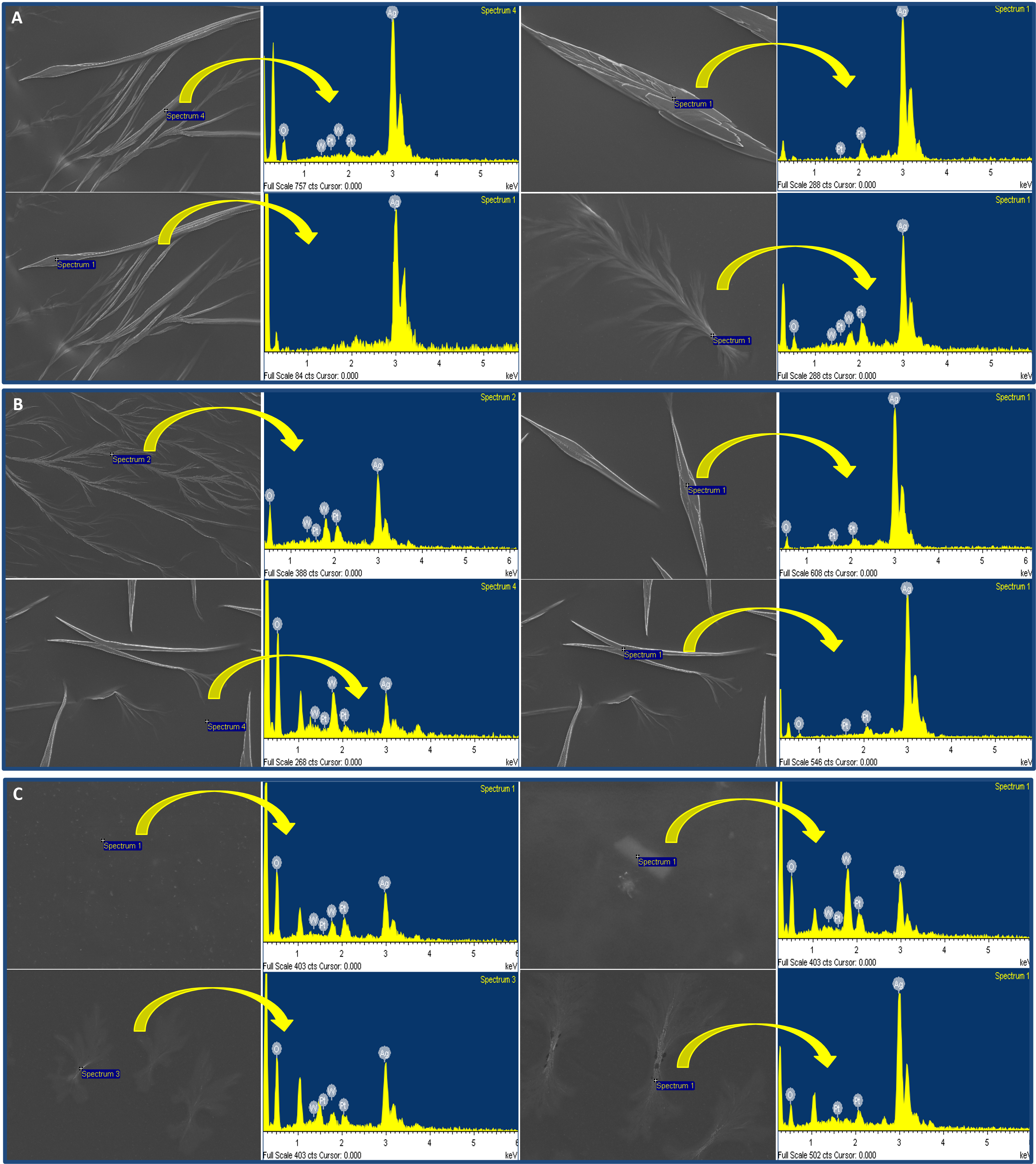


Figure S5. FE-SEM images of (A) CS3.3/α-Ag_2_WO_4_ (B) CS6.6/α-Ag_2_WO_4_ and (C) CS9.9/α-Ag_2_WO_4_ composites irradiated by fs laser, and EDS analysis.


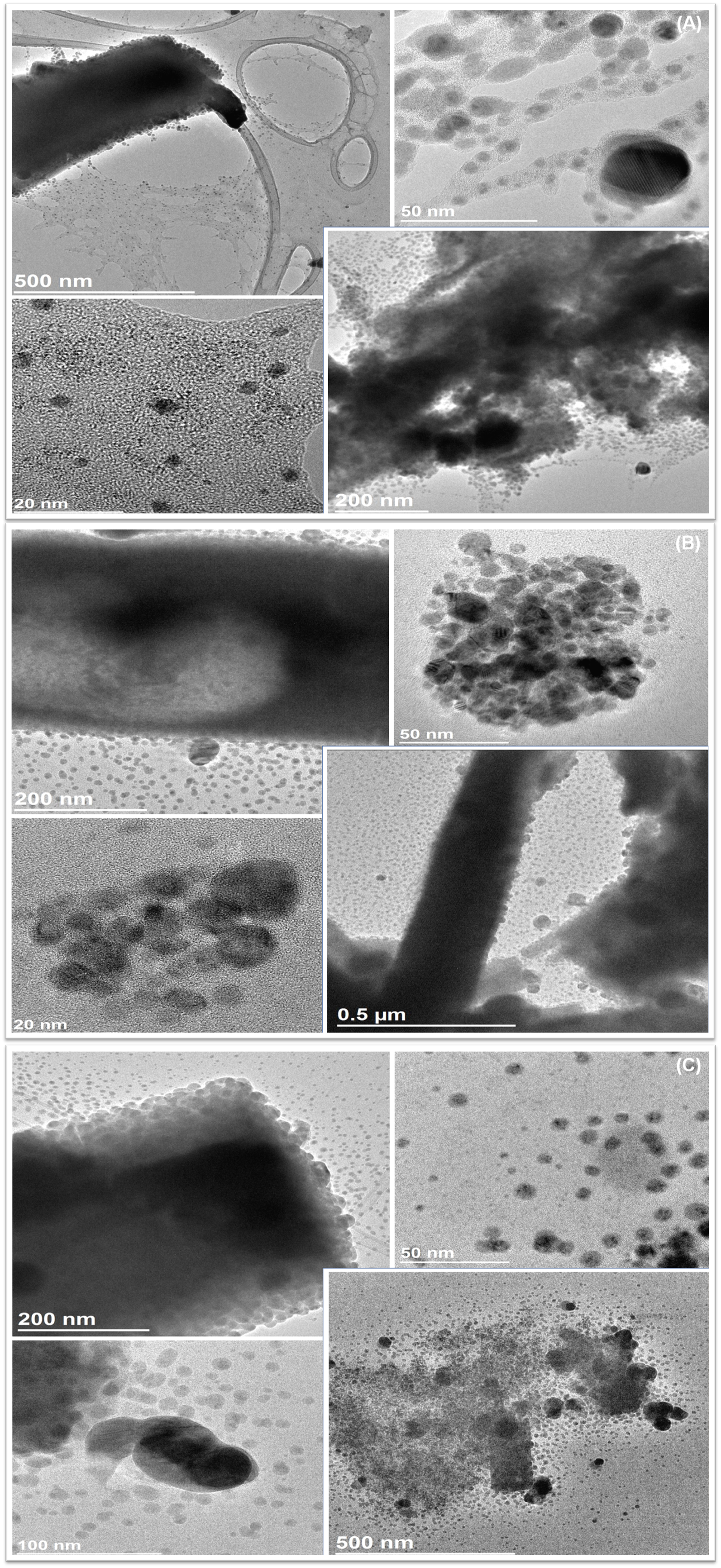


Figure S6. TEM and HR-TEM images of (A) CS3.3/α-Ag_2_WO_4_ (B) CS6.6/α-Ag_2_WO_4_ and (C) CS9.9/α-Ag_2_WO_4_ composites irradiated by fs laser.


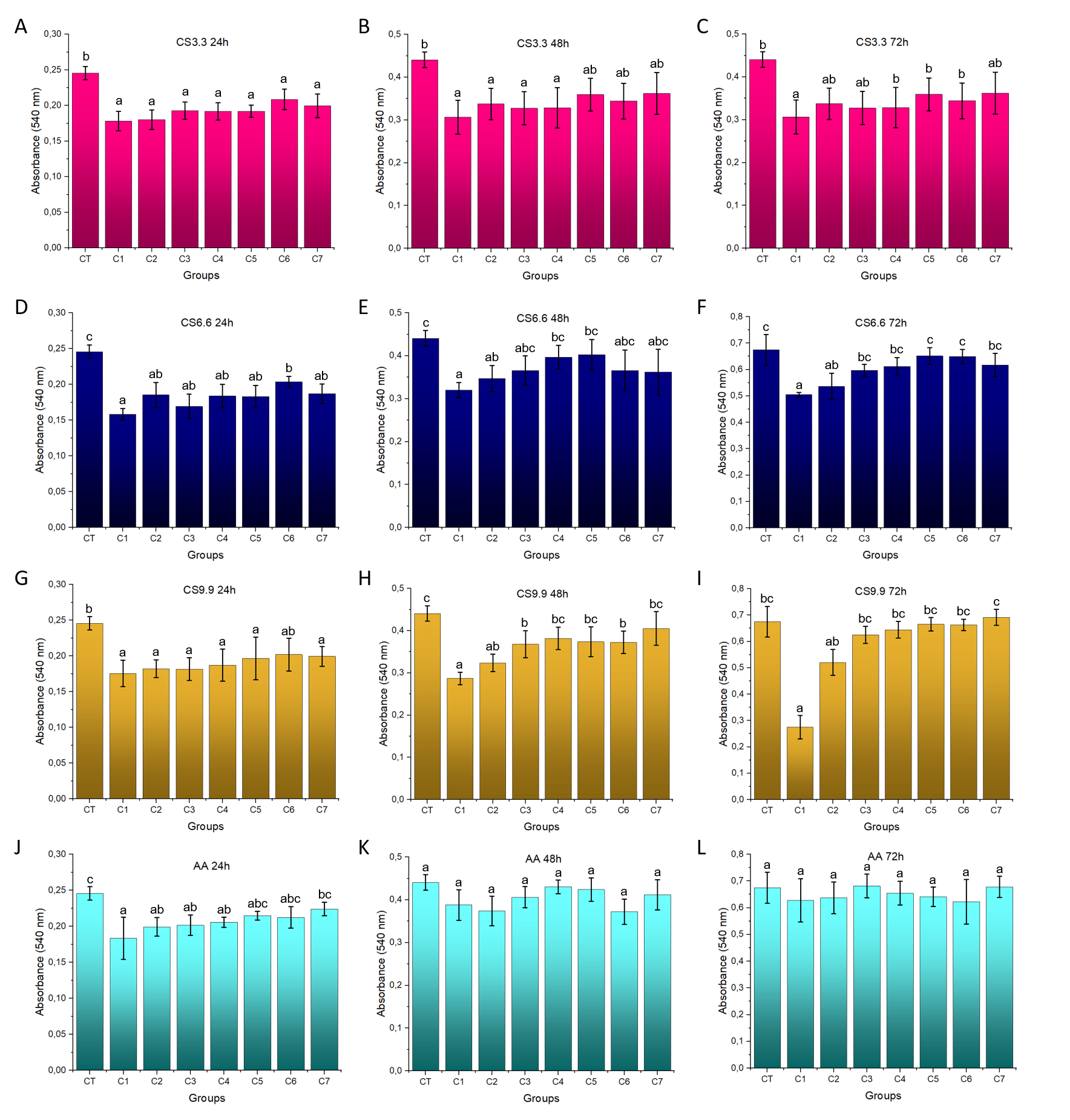


**Figure S7.** Cytotoxicity profile by MTT assay. Mean absorbance values after 24 h (A, D, G, J), 48 h (B, E, H, K) and 72 h (C, F, I, L) of treatment with different dilutions (C1 to C7) of vehicles: CS3.3 (A, B, C), CS6.6 (D, E, F), CS9.9 (G, H, I) and AA (J, K, L). CT: control; CS: chitosan; AA: acetic acid; C1: initial solution, C7: last dilution. Different letters denote statistically significant differences between groups (n = 12).


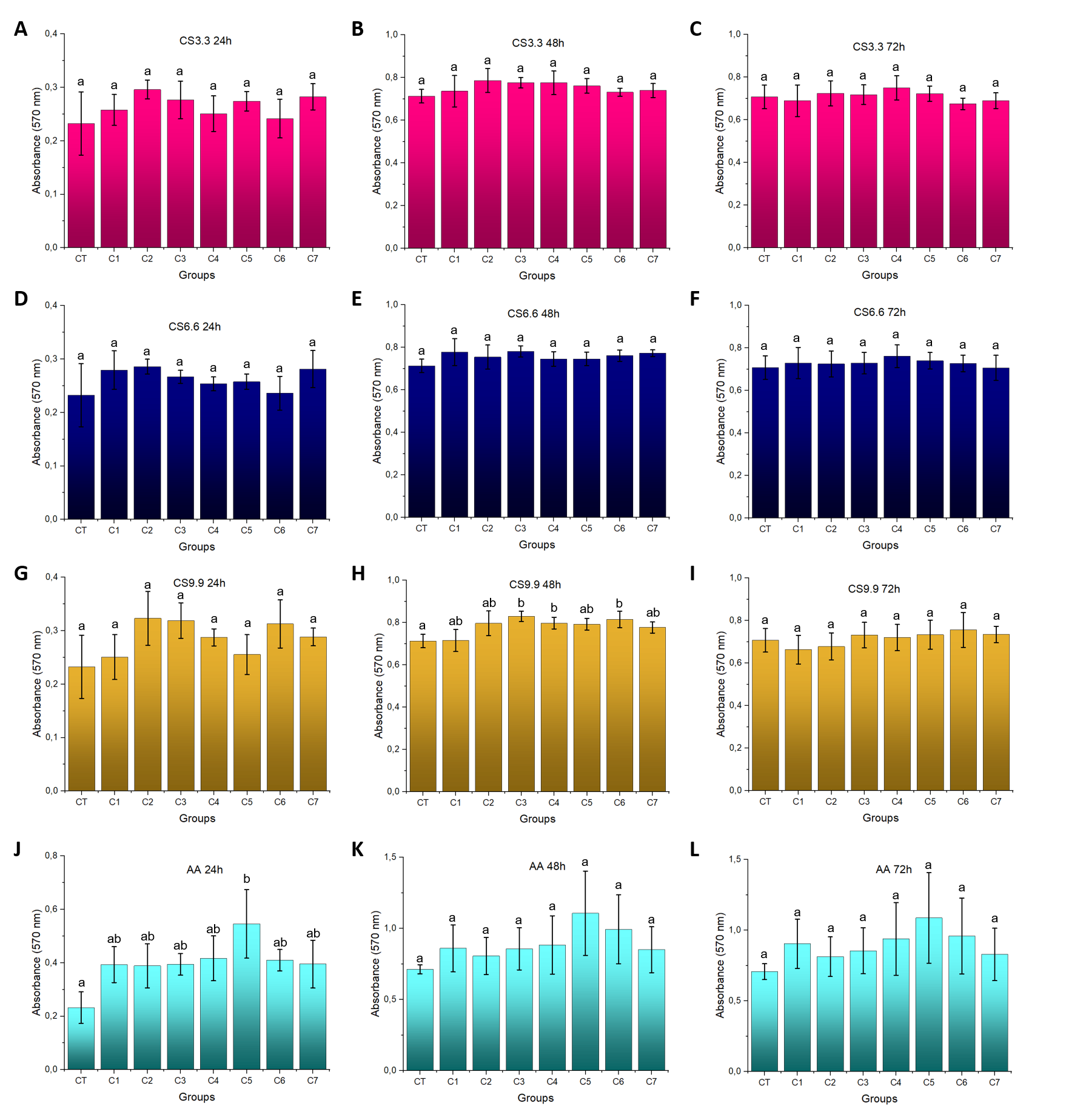


**Figure S8.** Cytotoxicity profile by Alamar Blue assay. Mean absorbance values after 24 h (A, D, G, J), 48 h (B, E, H, K) and 72 h (C, F, I, L) of treatment with different dilutions (C1 to C7) of vehicles: CS3.3 (A, B, C), CS6.6 (D, E, F), CS9.9 (G, H, I) and AA (J, K, L). CT: control; CS: chitosan; AA: acetic acid; C1: initial solution, C7: last dilution. Different letters denote statistically significant differences between groups (n = 12).

Table S1. FTIR wavenumber of CS3.3, CS6.6 and CS9.9 polymers and CS3.3/α-Ag_2_WO_4_, CS6.6/α-Ag_2_WO_4_ and CS9.9/α-Ag_2_WO_4_ composites irradiated by fs laser.

| 4000–2400 cm^-1^ | | | | | |
| --- | --- | --- | --- | --- | --- |
| Wavenumber (cm^-1^) | | | | | |
| 3.3, 6.6 and 9.9 g/L | 3359 | 3258 | 3097 | 2929 | 2875 |
| CS/α-Ag_2_WO_4_ composites | | | | | |
| (3.3 g/L) | - | 3260 | - | 2996 | 2925 |
| (6.6 g/L) | - | 3256 | - | 2925 | 2867 |
| (9.9 g/L) | - | 3256 | - | 2925 | 2867 |

| 2400–1200 cm^-1^ | | | | | |  |
| --- | --- | --- | --- | --- | --- | --- |
| Wavenumber (cm^-1^) | | | | | |  |
| 3.3, 6.6 and 9.9 g/L | 1636 | 1542 | 1404 | 1377 | 1324 | 1258 |
| CS/α-Ag_2_WO_4_ composites | | | | | |  |
| (3.3 g/L) | 1645 | 1514 | 1400 | 1379 | 1313 | 1251 |
| (6.6 g/L) | 1645 | 1551 | 1403 | 1375 | 1313 | 1251 |
| (9.9 g/L) | 1645 | 1540 | 1401 | 1390 | 1313 | 1242 |

| 1200–800 cm^-1^ | | | | | |  |
| --- | --- | --- | --- | --- | --- | --- |
| Wavenumber (cm^-1^) | | | | | |  |
| 3.3, 6.6 and 9.9 g/L | 1153 | 1063 | 1022 | 943 | 902 | - |
| CS/α-Ag_2_WO_4_ composites | | | | | |  |
| (3.3 g/L) | 1152 | 1070 | 1029 | 948 | 929 | 870 |
| (6.6 g/L) | 1150 | 1062 | 1022 | 946 | 920 | 867 |
| (9.9 g/L) | 1150 | 1060 | 1017 | 945 | 920 | 867 |

|  |  | |  | |  | | 800–50 cm^-1^ | | | | | |  |  |  |  |  |
| --- | --- | --- | --- | --- | --- | --- | --- | --- | --- | --- | --- | --- | --- | --- | --- | --- | --- |
|  |  | |  | |  | | Wavenumber (cm^-1^) | | | | | |  |  |  |  |  |
| 3.3, 6.6 and 9.9 g/L | - | | | 653 | | 617 | 558 | - | - | 323 | - | 209 | - |  |  | - |  |
|  |  | |  | |  | | CS/α-Ag_2_WO_4_ composites | | | | | |  |  |  |  |  |
| (3.3 g/L) | | 750 | | 664 | | 616 | 563 | 432 | 371 | 332 | 279 | 209 | 170 | - | - | 95 | - |
| (6.6 g/L) | | 743 | | 642 | | 630 | 571 | 433 | 371 | 335 | 268 | 208 | 168 | 142 | 112 | - | 64 |
| (9.9 g/L) | | 760 | | 660 | | 633 | 552 | 426 | 371 | 332 | 278 | 208 | 173 | 140 | 115 | 86 | 59 |

# References

1 Maria Mucha & Pawlak, A. Complex study on chitosan degradability. *Polimery* **47**, 509-516 (2002).

2 A. Sionkowska, A. Płanecka, K. Lewandowska, B. Kaczmarek & Szarszewska, P. Influence of UV-irradiation on molecular weight of chitosan. *Progress on Chemistry and Application of Chitin and its Derivatives* **XVIII**, 21-28 (2013).

3 Usman, M. S., Ibrahim, N. A., Shameli, K., Zainuddin, N. & Yunus, W. M. Copper nanoparticles mediated by chitosan: synthesis and characterization via chemical methods. *Molecules* **17**, 14928-14936, doi:10.3390/molecules171214928 (2012).

4 Anthony L. Andrady, Torikai, A. & Kobatake, T. Spectral Sensitivity of Chitosan Photodegradation. *Journal of Applied Polymer Science* **62**, 1465-1471 (1996).

5 P. H. D. Ferreira *et al.* Femtosecond laser induced synthesis of Au nanoparticles mediated by chitosan. *Optics Express* **20**, 518-523 (2011).

6 Khan, A. *et al.* Structural and antimicrobial properties of irradiated chitosan and its complexes with zinc. *Radiation Physics and Chemistry* **91**, 138-142, doi:10.1016/j.radphyschem.2013.05.025 (2013).

7 Vicentini, D. S., Smania, A. & Laranjeira, M. C. M. Chitosan/poly (vinyl alcohol) films containing ZnO nanoparticles and plasticizers. *Materials Science and Engineering: C* **30**, 503-508, doi:10.1016/j.msec.2009.01.026 (2010).

8 Li, L. H., Deng, J. C., Deng, H. R., Liu, Z. L. & Xin, L. Synthesis and characterization of chitosan/ZnO nanoparticle composite membranes. *Carbohydrate research* **345**, 994-998, doi:10.1016/j.carres.2010.03.019 (2010).

9 Tiwari, A. D., Mishra, A. K., Mishra, S. B., Kuvarega, A. T. & Mamba, B. B. Stabilisation of silver and copper nanoparticles in a chemically modified chitosan matrix. *Carbohydr Polym* **92**, 1402-1407, doi:10.1016/j.carbpol.2012.10.008 (2013).

10 P. F. S. Pereira *et al.* α-Ag_2-2x_Zn_x_WO_4_ (0 ≤ x ≤ 0.25) solid solutions: structure, morphology, and optical properties. *Inorg. Chem.* **56**, 7360-7372, doi:10.1021/acs.inorgchem.7b00201 (2017).

11 Zhaoyong Lin *et al.* Electronic Reconstruction of α‑Ag_2_WO_4_ Nanorods for Visible-Light Photocatalysis. *ACS Nano* **9**, 7256–7265 (2015).

12 Sreedevi, A. *et al.* Chemical synthesis, structural characterization and optical properties of nanophase α-Ag_2_WO_4_. *Indian J. Phys.* **89**, 889-897, doi:10.1007/s12648-015-0664-1 (2015).

13 Pereira, W. d. S. *et al.* Effects of chemical substitution on the structural and optical properties of α-Ag_2−2x_Ni_x_WO_4_ (0 ≤ x ≤ 0.08) solid solutions. *Phys. Chem. Chem. Phys.* **18**, 21966-21975, doi:10.1039/C6CP00575F (2016).

14 Thresiamma George, Sunny Joseph & Mathew, S. Synthesis and characterization of nanophased silver tungstate. *J. Phys.* **65**, 793–799 (2005).

15 Ramezani, M., Pourmortazavi, S. M., Sadeghpur, M., Yazdani, A. & Kohsari, I. Silver tungstate nanostructures: electrochemical synthesis and its statistical optimization. *J. Mater. Sci.-Mater. El.* **26**, 3861-3867, doi:10.1007/s10854-015-2912-8 (2015).
